# A Turbo‐Charging System‐Like Contrast Agent for MRI‐Guided STING Pathway‐Activated Cancer Immunotherapy

**DOI:** 10.1002/advs.202410432

**Published:** 2024-11-03

**Authors:** Bin Ren, Sihua Yan, Zongheng Li, Ya Huang, Haobin Cai, Jing Yang, Qingdeng Fan, Chunmei Chen, Fanchao Que, Guochao Wu, Lin Huang, Ruilong Zhou, Jiaoyang Zhu, Chenggong Yan, Gang Liu, Zheyu Shen, Shipeng Ning

**Affiliations:** ^1^ School of Biomedical Engineering Southern Medical University 1023 Shatai South Road Guangzhou Guangdong 510515 China; ^2^ Department of Breast Surgery The Second Affiliated Hospital of Guangxi Medical University Nanning 530000 China; ^3^ Medical Imaging Center Nanfang Hospital Southern Medical University 1023 Shatai South Road Guangzhou Guangdong 510515 China; ^4^ State Key Laboratory of Molecular Vaccinology and Molecular Diagnostics Center for Molecular Imaging and Translational Medicine School of Public Health Xiamen University Xiamen Fujian 361102 China

**Keywords:** cancer immunotherapy, Gd‐based contrast agents (GBCAs), magnetic resonance imaging (MRI), stimulator of interferon genes (STING), turbo‐charging system‐like contrast agent (Turbo S)

## Abstract

To overcome the problems of Gd‐based contrast agents (GBCAs) (nephrotoxicity and brain deposition) and stimulator of interferon genes (STING) agonists (poor stability, low delivery efficiency, and potential toxicity), in this study, a Turbo‐charging system‐like GBCA is designed and constructed for magnetic resonance imaging (MRI) guided STING pathway‐activated cancer immunotherapy. Poly(acrylic acid) (PAA) is used to coordinate with Gd^3+^, forming a Gd/PAA macrochelate. Both Gd/PAA macrochelate and SR717 are conjugated to cystamine (CA) to obtain SR717‐CA@Gd/PAA self‐assembled nanoparticles (SAN), which are termed as *Turbo S* because of its similarity with the Turbo‐charging system of cars. After accumulation in tumors and internalization in tumor cells, the disulfide linkage in *Turbo S* undergoes a cleavage process catalyzed by glutathione (GSH), leading to the release of Gd/PAA and SR717. The released Gd/PAA gain a high *r*_1_ value (17.11 mm ^−1^ s^−1^ at 7.0 T; 57.81 mm ^−1^ s^−1^ at 3.0 T), indicating its strong *T*_1_ imaging capability. *Turbo S* with a low dosage of SR717 (8.9 mg kg^−1^) achieved a higher tumor immunotherapeutic efficacy than free SR717 with a high dosage (30 mg kg^−1^). The excellent delivery efficiency, high tumor treatment efficacy, and superior biosafety demonstrate that the *Turbo S* can be used as a promising candidate for tumor immunotherapy.

## Graphical abstract

To overcome the problems of Gd‐based contrast agents (GBCAs) and stimulator of interferon genes (STING) agonists, in this study, a Turbo‐charging system‐like GBCA termed as *Turbo S* are designed and constructed for MRI‐guided STING pathway‐activated cancer immunotherapy.

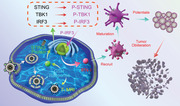

## 1 Introduction

Magnetic resonance imaging (MRI) has gained wide use in clinics due to its unparalleled spatial resolution, exquisite soft tissue contrast, non‐invasive nature, and commendable safety profile. It has become the cornerstone of disease diagnosis, particularly in the realm of early tumor detection and preoperative tumor evaluation. MRI enables non‐invasive and quantitative assessment of the efficacy of anti‐tumor drugs, providing a basis for adjusting treatment plans. Furthermore, it can visualize the release and distribution of drugs within target tissues, offering theoretical insights for the development of novel targeted carriers. Despite its pivotal role, MRI as a standalone imaging modality faces a problem of limited contrast between tumors and surrounding tissues. Hence, a number of MRI contrast agents (CAs), especially Gd‐based CAs (GBCAs), have been meticulously exploited to overcome this problem.^[^ ^1^ ^]^ These CAs intricately modulate signal intensity or elicit specific signal responses within the body's tissues to conspicuously delineate tumor regions. Although the GBCAs have been dominating the market of MRI CAs, they need high injection dosage of Gd (15.7 mg kg^−1^) to obtain clear tumor images because they lack tumor targetability,^[^ ^2^ ^]^ resulting in problems of nephrotoxicity and brain deposition warned by united states food and drug administration (U.S. FDA).

Immunotherapy, e.g., immune checkpoint blockade (ICB) therapy and chimeric antigen receptor (CAR) T cell therapy, has brought about revolutionary changes in cancer treatment and has been incorporated into treatment guidelines for various cancer types.^[^ ^3^ ^]^ One research area of immunotherapy is identifying targets that may trigger or enhance anti‐tumor immune responses, e.g., the stimulator of interferon genes (STING)^[^ ^4^ ^]^ STING plays a pivotal role as a signaling molecule in orchestrating the innate immune response within the body. The cytoplasmic deoxyribonucleic acid (DNA) sensor cyclic guanosine monophosphate (GMP) – adenosine monophosphate (AMP) synthase (cGAS) recognizes cytosolic tumor DNA, and then produces 2′3’‐cyclic GMP‐AMP (2′3’‐cGAMP), a natural ligand of the STING protein. The 2′3’‐cGAMP can bind to the homodimer of the adaptor protein STING on the endoplasmic reticulum (ER) membrane, forming a polymeric STING architecture. Upon conformational changes in the STING protein, activation occurs triggering a cascade signaling involving TANK binding kinase 1 (TBK1) and downstream interferon (IFN) regulatory factor 3 (IRF3), which results in the production of type I interferons (IFN‐I).^[^ ^5^ ^]^ The IFN‐I recruits immature dendritic cells (DCs) and promotes their maturation, thereby enhancing CD8^+^ T cell expression. IFN‐γ is secreted by CD8^+^ T cells, synergizing with natural killer (NK) cells to exert potent anti‐tumor effects.

The natural ligand of STING, cyclic dinucleotides (CDN), is one of the famous STING agonists. However, as a hydrophilic small molecule with poor targetability, CDN is not suitable for systemic administration because of the limited intratumor delivery and degradation by numerous nucleotide enzymes in the body.^[^ ^6^ ^]^ Other reported STING agonists (e.g., MSA‐2, SR717, and MK‐1454) were continuously developed to address the shortcomings of CDN.^[^ ^7^ ^]^ SR717 is a 2′3’‐cGAMP mimic that can bind to the same site on STING, inducing the same conformational changes in STING. However, these small molecular STING agonists face toxicity issues, and they are difficult to accumulate in the tumor microenvironment (TME), which limit the clinical translation of them.^[^ ^7^ ^]^ Overall, targeted immunotherapy using STING agonists remains a big challenge of poor stability, low delivery efficiency, and potential toxicity.

**Scheme 1:**
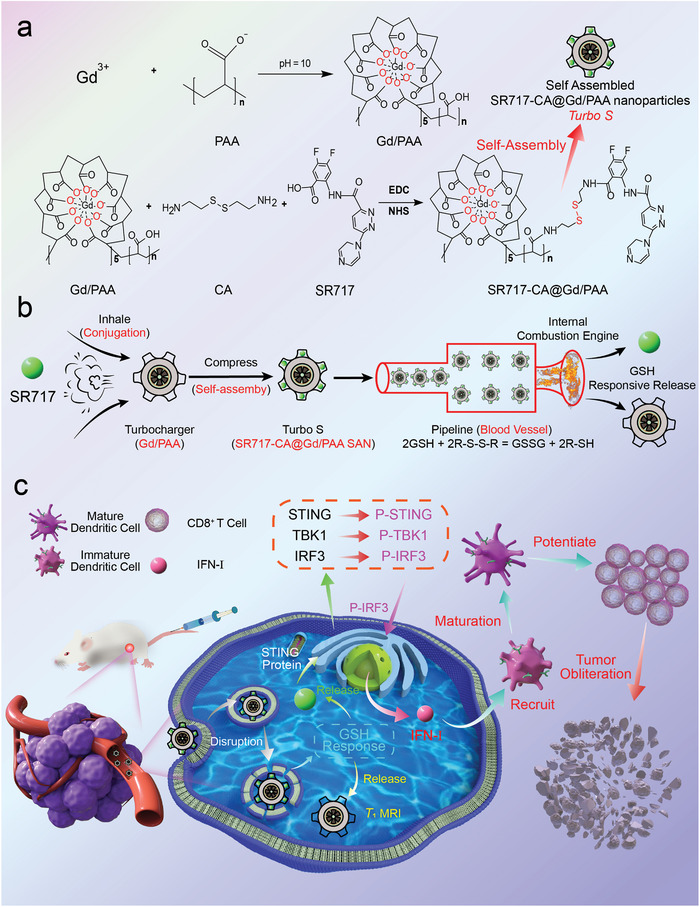
a). Schematic illustration for the synthesis of Gd/PAA, SR717‐CA@Gd/PAA, and self‐assembled SR717‐CA@Gd/PAA nanoparticles (i.e., Turbo‐charging system‐like contrast agent, *Turbo S*). b): Schematic illustration for similarity between the *Turbo S* and Turbo‐charging system in cars. c): Mechanism of the *Turbo S* for MRI‐guided STING pathway‐activated cancer immunotherapy.

It is reported that the activation of STING in cancer cells does not contribute to the therapeutic effect, but the tumor tissues can produce CDN that transfers to tumor‐associated DCs and macrophages, and induces the production of IFN‐I, thereby activating the STING pathway of host immune cells.^[^ ^6^ ^]^ It is also reported that the signal of STING in tumor cells is inhibited due to epigenetic silencing or missense mutations of STING or its upstream regulator cGAS.^[^ ^8^ ^]^ However, when the tumor also expresses STING, all CDN‐based and non‐nucleotide STING agonists can yield optimal outcomes. And the key to the tumor immune killing effect mediated by the STING pathway lies in the secretion of IFN‐I.^[^ ^6^ ^]^ And SR717 is a mimic of CDN, which can induce the presentation of tumor molecules to the immune system and activate toxic T cells and NK cells within tumors and draining lymph nodes.^[^ ^7^ ^]^ So, to overcome the aforementioned challenges associated with GBCAs and STING agonists, a Turbo‐charging system‐like GBCA, termed as *Turbo S*, was designed and constructed for MRI‐guided STING pathway‐activated cancer immunotherapy. As shown in **Scheme** **1a**, poly(acrylic acid) (PAA) coordinates with Gd^3+^, forming a Gd/PAA macrochelate. Both Gd/PAA macrochelate and SR717 are respectively conjugated to the two terminal amino groups of cystamine (CA), generating SR717‐CA@Gd/PAA macromolecules. A structure is formed with Gd/PAA exposed on the outer layer and SR717 encapsulated in the inner layer. The self‐assembly of SR717‐CA@Gd/PAA macromolecules results in the generation of SR717‐CA@Gd/PAA self‐assembled nanoparticles (SAN), which is termed as *Turbo S* because of its similarity with the Turbo‐charging system of cars.

The similarity between the *Turbo S* and the Turbo‐charging system of cars is shown in Scheme 1b. SR717 (compared to air) is conjugated onto (inhaled into) the Gd/PAA macrochelate (compared to turbocharger of cars) via the CA linker. The obtained SR717‐CA@Gd/PAA macromolecules can be self‐assembled (compressed) to SR717‐CA@Gd/PAA SAN (i.e., *Turbo S*). After blood circulation (fluxion in the pipeline) and tumor accumulation, the SR717‐CA@Gd/PAA SAN responds to the over‐expressed glutathione (GSH) in the TME and then releases SR717 and Gd/PAA macrochelate. The increase of SR717 accumulation in tumors for higher immunotherapeutic efficacy is very similar to the enhancement of compressed air inhaled in the internal combustion engine of cars for higher output power.

As shown in Scheme 1c, after accumulation in tumors and internalization in tumor cells, the GSH‐mediated cleavage of disulfide bond (─S─S─) in *Turbo S* facilitates the release of Gd/PAA and SR717. Although the *Turbo S* itself is a good *T*_1_‐weighted contrast agent for MRI with a high longitudinal relaxivity (*r*_1_ value) (7.55 mm ^−1^ s^−1^ at 7.0 T; 27.86 mm ^−1^ s^−1^ at 3.0 T), the released Gd/PAA gain a higher *r*_1_ value (17.11 mm ^−1^ s^−1^ at 7.0 T; 57.81 mm ^−1^ s^−1^ at 3.0 T), enhancing the *T*_1_ imaging capability. In addition, the half‐life of *Turbo S* is 5.52 h, which is significantly longer than that of Gd/PAA (15 min), resulting in a profoundly prolonged imaging time window.^[^ ^9^ ^]^ Moreover, the release of Gd/PAA and SR717 from *Turbo S* in TME takes time, which is similar to the lag of turbocharger in vehicles.^[^ ^10^ ^]^ SR717 released from *Turbo S* binds to the STING protein on the ER, causing the phosphorylation of STING (P‐STING).^[^ ^11^ ^]^ P‐STING leads to the phosphorylation of TBK1 (P‐TBK1), which in turn phosphorylates IRF3 (P‐IRF3).^[^ ^12^ ^]^ P‐IRF3 then enters the nucleus, resulting in the production of type I interferons (IFN‐I). The IFN‐I recruits immature DCs and promote their differentiation into mature DCs, which facilitate the expression of CD8^+^ T cells and NK cells, ultimately achieving bodacious innate immune‐mediated tumor cell killing.

## 2 Results and Discussion

### 2.1 Characterization of *Turbo S*

SR717 was first conjugated onto one terminal amino group of CA, generating SR717‐CA. SR717‐CA1‐6@Gd/PAA SAN were then synthesized with different molar ratios of SR717‐CA to Gd/PAA, and the synthesis conditions and characterization results are summarized in Table S1 (Supporting Information). The *T*_1_‐ or *T*_2_‐weighted MR images of SR717‐CA1‐6@Gd/PAA SAN and Gd/PAA measured on a 3.0 T clinical MRI scanner (Figure S1a, or Figure S2a, Supporting Information) show stronger *T*_1_ or *T*_2_ signal at higher *C*_Gd_. The *r*_1_ or *r*_2_ values of SR717‐CA1‐6@Gd/PAA SAN and Gd/PAA (3.0 T) can be obtained from the slopes of fitted lines in Figure S1b, or Figure S2b (Supporting Information). Compared with SR717‐CA1‐3@Gd/PAA SAN, SR717‐CA4@Gd/PAA SAN exhibits much higher Gd recovery (89.8%), SR717 loading content (13.3%), and *r*_1_ value (27.86 mM^−1^ s^−1^, at 3.0 T), but a lower *r*_2_/*r*_1_ ratio (2.27, at 3.0 T). Compared with SR717‐CA5,6@Gd/PAA SAN, SR717‐CA4@Gd/PAA SAN possesses much higher SR717 loading content and a lower *r*_2_/*r*_1_ ratio. Therefore, SR717‐CA4@Gd/PAA SAN as the optimal sample can be used as the *Turbo S* for STING pathway activation and *T*_1_ imaging.

**Figure 1:**
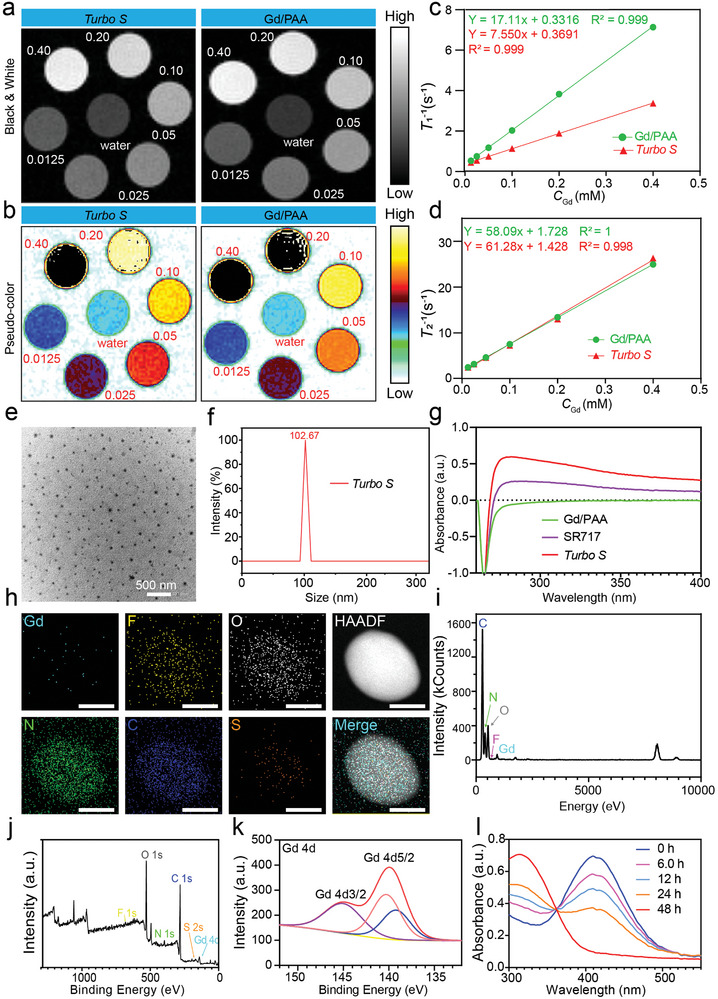
Physical characterization of *Turbo S*. a,b): *T*_1_‐weighted grayscale images (a), the corresponding pseudo‐colored images (b) of *Turbo S*, and Gd/PAA at different concentrations observed on a 7.0 T MRI scanner. c,d): *T*_1_ relaxation rate (1/*T*_1_) (c) or *T*_2_ relaxation rate (1/*T*_2_) (d) plotted as a function of *C*_Gd_ for *Turbo S*, and Gd/PAA measured at 7.0 T. e): TEM image of *Turbo S*. f): Hydrodynamic size distributions of *Turbo S*. g): UV–Vis absorption spectra of Gd/PAA, SR717, and *Turbo S* dissolved in DMF. h,i): Elemental mapping (h), and Energy‐dispersive X‐ray spectroscopy (EDS) (i) of *Turbo S* with peaks corresponding to Gd, F, O, N, C, and S. Scale bar: 30 nm. J,k): X‐ray photoelectron spectroscopy (XPS) of *Turbo S* with peaks corresponding to Gd 3d, F 1s, O 1s, N 1s, C 1s, and S 2s (j), and high‐resolution XPS spectra of Gd 4d of *Turbo S* (k). l): UV–vis spectra of *Turbo S* solutions measured by the DTNB assay at pH 7.4 after various incubation durations (0, 6.0, 12, 24, or 48 h) with 5.0 mm of GSH.

*T*_1_‐weighted (**Figure** **1**a,b) and *T*_2_‐weighted MR images (Figure S3a,b, Supporting Information) of *Turbo S* and Gd/PAA measured on a 7.0 T MRI scanner reinforce the strong concentration gradient dependence of MRI signals. The *r*_1_ and *r*_2_ values of *Turbo S* and Gd/PAA at 7.0 T are obtained from the slopes of fitted lines in Figure 1c,d. Although the *r*_1_ of *Turbo S* (7.55 mm ^−1^ s^−1^, 7.0 T) is lower than that of Gd/PAA (17.11 mm ^−1^ s^−1^, 7.0 T), it still outperforms commercially available contrast agent Gadovist (*r*_1_: 3.77 mm ^−1^ s^−1^, 7.0 T). Therefore, the *Turbo S* retains good *T*_1_‐MRI capabilities,^[^ ^8^ ^]^ allowing for a significant reduction in the clinical MRI dose of Gd, thereby reducing the risk of nephrotoxicity (nephrogenic systemic fibrosis) and Gd deposition in the brain. The 3.0 T MRI scanner, owing to its prevalent utilization in hospitals, serves as a prime exemplar for assessing the behavior of the material in authentic clinical settings. While the 7.0 T scanner, renowned for its exceptional field strength, offers superior image clarity and resolution, its clinical application is limited. The heightened magnetic field of the 7.0 T scanner, in comparison to the 3.0 T model, intensifies MRI signal strength and elevates the ΔSNR metric. Consequently, both 3.0 T and 7.0 T MRI systems are employed to comprehensively evaluate material performance.

The image of *Turbo S* and Gd/PAA solutions under laser irradiation (Figure S4, Supporting Information) show that the Tyndall effect is observed for *Turbo S*, but not for Gd/PAA, which indicates that the *Turbo S* solution is a colloid. To confirm this phenomenon, transmission electron microscopy (TEM) is taken advantage of to observe the morphology and distribution of *Turbo S* (Figure 1e). *Turbo S* gains excellent water dispersibility and its mean particle size measured by TEM is 53.2 nm (Figure S5, Supporting Information). The hydrodynamic diameter of *Turbo S* is 102.67 nm measured by dynamic light scattering (DLS) (Figure 1f), which exceeds the size obtained by TEM. That's because the organic molecules of *Turbo S* swell in pure water, while the TEM provides the real size in a dry state.

Characteristic peaks of 3410 cm^−1^ (O─H stretching vibration in carboxyl groups), 1712 cm^−1^ (stretching vibration peak of benzene rings), 1643 cm^−1^ (C═O stretching vibration of amide bonds), 1280 cm^−1^ (C─N stretching vibration of amide bonds), and 430 cm^−1^ (stretching vibration of disulfide bonds) are observed in the Fourier‐transform infrared (FT‐IR) spectrum of *Turbo S* (Figure S6, Supporting Information), indicating the successful synthesis of SR717‐CA4@Gd/PAA in *Turbo S*.^[^ ^13^ ^]^

Moreover, The zeta potential on the surface of *Turbo S* is −35.83 mv, lower than that of Gd/PAA (−12.11 mV) (Figure S7, Supporting Information). Zeta potential refers to the potential difference between the stern layer and the shear layer. *Turbo S* is self‐assembled from multiple SR717‐CA@GdPAA units, forming a nanoparticle structure with Gd/PAA on the exterior and SR717 on the interior. The negative charges on Gd/PAA are imparted by the carboxyl groups on its surface. Compared with Gd/PAA alone, the distance between the stern layer and the shear layer of *Turbo S* increases, resulting in a larger potential difference and thus a more negative zeta potential. Nanoparticles with a higher negative charge contribute to robust electrostatic repulsion, preventing aggregation in water and enhancing their dispersibility. Moreover, the nanoparticles with a positive charge are more likely to disrupt the cell membrane structure of normal cells, exhibiting cytotoxic effects before encountering tumor cells because most normal and tumor cells carry a negative charge.^[^ ^14^ ^]^

The particle size of *Turbo S* was also measured in water or Dulbecco's modified eagle medium (DMEM) + 10% fetal bovine serum (FBS) solution by DLS after storage at room temperature for 1.0–60 days (Figure S8, Supporting Information). The hydrodynamic diameter of *Turbo S* shows almost no change in both solutions, which indicates the brilliant stability of *Turbo S*. The excellent water dispersibility and stability exhibited by *Turbo S* can be attributed to the negative charge, resulting in strong electrostatic repulsion among them.

The ultraviolet absorption characteristics of Gd/PAA, *Turbo S*, and SR717 (Figure 1g) show that the *Turbo S* and SR717 exhibit similar absorption wavelength (269 nm), which indicates the successful conjugation of SR717 in *Turbo S*. The high‐angle annular dark‐field scanning transmission electron microscopy (HAADF‐STEM, Figure 1h) and energy‐dispersive X‐ray (EDX) spectroscopy (Figure 1i) show the existence of Gd, F, O, N, C, and S elements in *Turbo S*, indicating the chemical composition of SR717‐CA@Gd/PAA macromolecules in *Turbo S*. Figure 1j displays the X‐ray photoelectron spectroscopy (XPS) spectrum of *Turbo S*, showing the characteristic peaks of O 1s, N 1s, C 1s, F 1s, S 2s, and Gd 4d. The XPS spectrum of Gd 4d exhibits two obvious peaks at 145.0 eV (Gd 4d3/2) and 140.0 eV (Gd 4d5/2) (Figure 1k), indicating the presence of Gd^3+^ in *Turbo S*.

Figure 1l shows ultraviolet‐visible (UV‐vis) spectra of *Turbo S* solutions measured by the 5,5′‐dithiobis (2‐nitrobenzoic acid) (DTNB) assay after incubation with GSH at pH 7.4 for different time to test the GSH depletion capacities (DTNB + GSH = TNB + GSSG). As the incubation time increases, more GSH is consumed by *Turbo S*, resulting in less TNB produced, indicating the time‐dependent GSH depletion by *Turbo S*. The GSH consumption means the breakdown of the disulfide bond in *Turbo S*, which leads to the continuous release of Gd/PAA and SR717 from *Turbo S*, potentially enabling MRI‐guided activation of the STING pathway for immunotherapy.

### 2.2 Characterization for the GSH Responsiveness of *Turbo S* In Vitro

**Figure 2:**
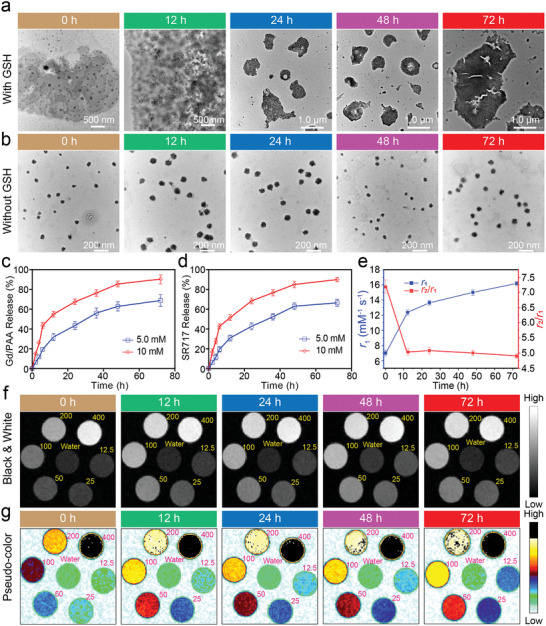
Characterization for the GSH responsiveness of *Turbo S* in vitro. a,b): TEM images of *Turbo S* after incubation in PBS at pH 7.4 with (a), or without (b) GSH (5.0 mm) for 0, 12, 24, 48, or 72 h. c,d): Release behaviors of Gd/PAA (c) or SR717 (d) from *Turbo S* in pH 7.4 PBS with 5.0, or 10 mm of GSH. Mean ± SD., *n* = 3. e): The changes of *r*_1_ value (blue curve) or *r*_2_/*r*_1_ ratio (red curve) of *Turbo S* measured on a 7.0 T MRI scanner after incubation with pH 7.4 of PBS containing 10 mm of GSH at 37 °C for 0–72 h. Mean ± SD, *n* = 3. f,g): The balck & whtie (f), and the pseudo‐colored (g) *T*_1_‐weighted images of *Turbo S* observed on a 7.0 T MRI scanner after incubation in PBS (pH 7.4) with 10 mm of GSH for different times.

*Turbo S* was incubated in phosphate‐buffered saline (PBS) with or without 5.0 mM of GSH (simulated TME)^[^ ^15^ ^]^ at 37 °C for different times (0–72 h) to evaluate the GSH responsiveness of *Turbo S*, whose morphologies were observed by TEM (**Figure** **2**a,b). The morphologies of *Turbo S* pre‐ and post‐incubation in PBS without GSH present dispersed nanoparticles, but post‐incubation in PBS with GSH show aggregated clusters. That is because the coordination between Gd^3+^ and PAA is very stable, the released content from *Turbo S* is Gd/PAA chelate, but not Gd^3+^.

The UV–vis absorption spectra of SR717 solutions in *N*,*N*‐dimethylformamide (DMF) with different concentrations were observed from 260 to 380 nm, and a standard curve is fitted based on the absorbance at 294 nm for each concentration (Figure S9a, b, Supporting Information). The SR717 loading content and loading efficiency of SR717‐CA1‐6@Gd/PAA SAN are measured based on the standard curve, calculated according to Equations (1) and (2), and summarized in Table S1 (Supporting Information).

(1) $$\mathrm{SR}717\;\;\mathrm{Loading}\;\;\mathrm{Content}=\frac{\mathrm{Mass}\;\;\mathrm{of}\;\;\mathrm{loading}\;\;\mathrm{SR}717}{\mathrm{Mass}\;\;\mathrm{of}\;\;\mathrm{Gd}/\mathrm{PAA}}\times 100\%$$

(2) $$\mathrm{SR}717\;\;\mathrm{Loading}\;\mathrm{Efficiency}=\frac{\mathrm{Mass}\;\;\mathrm{of}\;\;\mathrm{loading}\;\;\mathrm{SR}717}{\mathrm{Mass}\;\;\mathrm{of}\;\;\mathrm{feeding}\;\;\mathrm{SR}717}\times 100\%$$

Figure 2c,d shows the release behaviors of Gd/PAA and SR717 from *Turbo S* in PBS 7.4 with 5.0 or 10 mm of GSH, tested by inductively coupled plasma‐optical emission spectrometer (ICP‐OES) or UV‐vis. Gd/PAA and SR717 are gradually released from *Turbo S* over time, and the release of Gd/PAA and SR717 accelerates with the increase of GSH concentration. The release of Gd/PAA and SR717 within 12 h respectively reaches 55.03 ± 2.93% and 51.41 ± 1.92% with 10 mm of GSH, owing to the presence of disulfide bonds within *Turbo S* that is highly responsive to GSH. These results demonstrate that the targeted GSH‐triggered degradation of *Turbo S* and the regulated drug release behavior have the potential to mitigate adverse effects on healthy tissues, thereby enhancing the biocompatibility of *Turbo S*.

The *Turbo S* solutions after 0–72 h of incubation in PBS (pH 7.4) with or without 10 mm of GSH are tested by a 7.0 T MRI (Figure S10 or Figure S11, Supporting Information), and the *r*_1_ and *r*_2_ values were obtained from the slopes of the fitted lines. The corresponding changes in *r*_1_ values and *r*_2_/*r*_1_ ratios are shown in Figure 2e or Figure S12 (Supporting Information). After incubation in PBS with 10 mm of GSH, the *r*_1_ of *Turbo S* significantly increases from 7.0 ± 0.54 to 16.17 ± 0.21 mm ^−1^ s^−1^, and the *r*_2_/*r*_1_ decreases from 7.27 ± 0.25 to 4.90 ± 0.07. However, after incubation in PBS without GSH, both *r*_1_ and *r*_2_/*r*_1_ of *Turbo S* show slight change.

The MR images of *Turbo S* measured on a 7.0 T MRI scanner after incubation in pH 7.4 of PBS with 10 mm of GSH (Figure 2f,g) become progressively brighter at the same concentration with increasing the incubation time, while that without GSH (Figure S13a,b, Supporting Information) remains almost unchanged.

The above results reinforce that our *Turbo S* can be degraded in the presence of GSH, and the GSH‐responsive release of Gd/PAA and SR717 from *Turbo S* also takes time, which is analogous to the lag observed in the turbocharging system of automobiles. Turbo lag refers to the delay in response of a turbocharged engine, where the turbocharging system delivers higher power after the initial lag.^[^ ^16^ ^]^ The activation of *T*_1_ MRI by GSH enhances its suitability for subsequent high‐contrast tumor imaging in living organisms.

### 2.3 Activation of STING and Downstream Signalling with *Turbo S*

**Figure 3:**
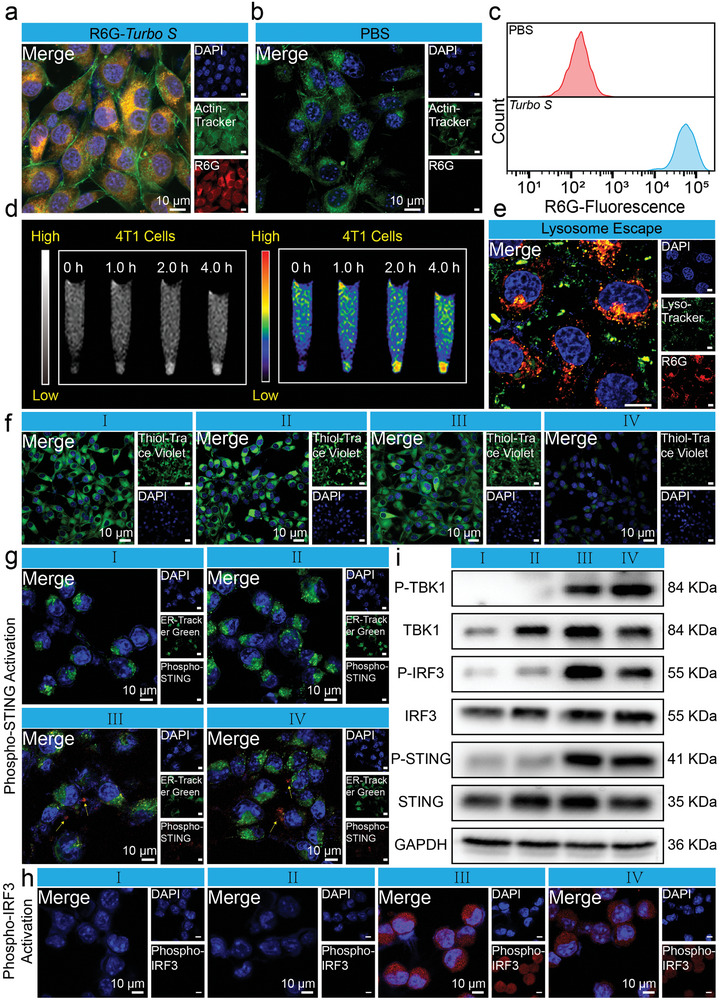
Interactions between 4T1 cells and *Turbo S*. a,b): Confocal laser scanning microscopy (CLSM) images of 4T1 cells after incubation with R6G‐*Turbo S* (red fluorescence) (a) or PBS (b) for 4.0 h (green fluorescence: Actin‐Tracker for cytoskeleton, blue fluorescence: DAPI for cell nuclei). c): Flow cytometry analysis of the distribution of R6G fluorescence in 4T1 cells after incubation with PBS (red), or R6G‐*Turbo S* (blue) for 4.0 h. d): The grayscale (left) and the colored (right) images of 4T1 cells after incubation with *Turbo S* for 0, 1.0, 2.0, and 4.0 h, observed on a 3.0 T clinical MRI. e): CLSM images of 4T1 cells after 8.0 h of treatment with R6G‐*Turbo S*, and staining with Lyso‐Tracker (green fluorescence for lysosomes) and DAPI. f): CLSM images of 4T1 cells after 24 h of treatment with group of PBS (I), Gd/PAA (II), free SR717 (III), or *Turbo S* (IV), and staining with Thiol–Trace Violet (green fluorescence for intracellular GSH) and DAPI. g): CLSM images of 4T1 cells after 24 h of treatment with the group I–IV (green fluorescence: ER‐Tracker Green for endoplasmic reticulum, red fluorescence: phospho‐STING for phosphorylated STING protein, blue fluorescence: DAPI). h): CLSM images of 4T1 cells after 24 h of treatment with the group I–IV (red fluorescence: phospho‐IRF3 for phosphorylated IRF3 protein, blue fluorescence: DAPI). Scale bar: 10 µm. i): Western blot analysis of expression level of P‐TBK1, TBK1, P‐IRF3, IRF3, P‐STING, STING in 4T1 cells after 24 h of treatment with the group I‐IV, using GAPDH as the internal control.

**Figures** **3**a and S14a (Supporting Information) exhibit confocal laser scanning microscopy (CLSM) images of mouse breast cancer cells 4T1 and mouse colon cancer cells MC38 cells, where the cell cytoskeleton and nucleus are stained with fluorescein isothiocyanate (FITC)‐phalloidin (green fluorescence) and 4′,6‐diamidino‐2‐phenylindole (DAPI, blue fluorescence), respectively. The cytoplasm of 4T1 or MC38 cells treated with Rhodamine 6G (R6G)‐*Turbo S* exhibits much more red fluorescence than that of group PBS (Figure 3b; Figure S14b, Supporting Information), which indicates that R6G‐loaded *Turbo S* can be uptaken by 4T1 and MC38 cells. The fluorescence distributions (Figure 3c; Figure S16a, Supporting Information) and the corresponding quantitative analysis (Figures S15 and Figure S16b, Supporting Information) of 4T1 and MC38 cells measured by flow cytometry show that the differences in fluorescence intensity between the group R6G‐*Turbo S* and the group PBS are statistically significant (^****^ *p* < 0.0001), indicating the effective internalization of *Turbo S* by tumor cells. The internalized amount of *Turbo S* in 4T1 or MC38 cells was subsequently determined by ICP‐OES (Figure S17, Supporting Information). The uptake of *Turbo S* by tumor cells escalates proportionally with extended incubation periods, with minimal discernible variation between 4T1 (2.82 ± 0.17 pg per cell) and MC38 (2.76 ± 0.29 pg per cell) cell lines.

The high uptake by tumor cells indicates that cell experiments can be utilized to evaluate the MRI efficiency of *Turbo S*. Afterward, 4T1 cells are incubated with *Turbo S* for various durations, and the cell images are observed by a 3.0 T clinical MRI scanner (Figure 3d). It is noteworthy that the MR signals of 4T1 cells increase over time due to the increased uptake of *Turbo S*, which demonstrates the strong MRI capability of *Turbo S*.

Because the internalized nanoparticles by cells are easily digested by lysosomes,^[^ ^17^ ^]^ the ability of nanoparticles to evade lysosomal degradation is pivotal in determining their fate. CLSM visualization of 4T1 cells post‐incubation with R6G‐*Turbo S* (Figure 3e) a clear separation between the intense red fluorescence (R6G‐*Turbo S*) and the green fluorescence region (lysosomes), demonstrating the successful evasion from lysosomal of *Turbo S*. That's because *Turbo S* obtains a multitude of tertiary amines that exhibit a proton sponge effect, increasing the osmotic imbalance in lysosomes. An influx of water causes lysosomal swelling and rupture, releasing *Turbo S* nanoparticles into the cytoplasm.^[^ ^18^ ^]^

After staining 4T1 cells with the GSH probe Thiol‐Trace Violet and visualizing them under CLSM (Figure 3f), a pronounced green fluorescence is observed in cells treated with PBS, Gd/PAA, or free SR717 (groups I–III). Conversely, the fluorescence intensity diminishes significantly in cells treated with *Turbo S* (group IV), indicative of the ability of *Turbo S* to deplete GSH in tumor cells, leading to the release of Gd/PAA and SR717. The quantitative analysis of intracellular GSH (Figure S18, Supporting Information) measured by GSH assay Kit shows that the normalized GSH levels within 4T1 cells are 100%, 101.48 ± 2.42%, 101.63 ± 3.34%, or 43.84 ± 3.52% after treatment with group I‐IV, respectively. The significant reduction in GSH levels observed in group IV is attributed to the presence of a disulfide bond in *Turbo S*, which is highly sensitive to GSH. This marked reduction underscores the potent capability of *Turbo S* in effectively depleting GSH levels within the cells.^[^ ^18^ ^]^

CLSM images (Figure 3g) of 4T1 cells incubated with I–IV groups for 24 h and stained with rabbit anti‐mouse P‐STING primary antibody and goat anti‐rabbit secondary antibody were used to verify the activation of STING in tumor cells. Red fluorescence is observed in the groups of free SR717 and *Turbo S* with limited co‐localization with green fluorescence, which indicates the STING activation in tumor cells owing to the migration of P‐STING protein from the ER to the Golgi apparatus.^[^ ^20^ ^]^

During the activation process of the STING pathway, P‐STING prompts the phosphorylation of TBK1, leading to the generation of P‐TBK1. Subsequently, P‐TBK1 facilitates the phosphorylated IRF3, resulting in the formation of P‐IRF3.^[^ ^21^ ^]^ CLSM images (Figure 3h) of 4T1 cells stained with rabbit anti‐mouse P‐IRF3 primary antibody and goat anti‐rabbit secondary antibody show strong red fluorescence within and around the nucleus after treatment with free SR717 (positive control) and *Turbo S*. However, the groups of Gd/PAA and PBS (negative control) do not exhibit red fluorescence. These results demonstrate that *Turbo S* can activate the downstream signaling of the STING pathway and generate P‐IRF3.

Western blot analysis with glyceraldehyde‐3‐phosphate dehydrogenase (GAPDH) protein serving as the internal reference protein for normalization (Figure 3i; Figure S19a–c, Supporting Information) was utilized to assess the expression levels of P‐STING, P‐TBK1, and P‐IRF3 in 4T1 cells. The groups treated with free SR717 and *Turbo S* exhibited much higher levels of P‐STING, P‐TBK1, and P‐IRF3 than that of the groups treated with PBS or Gd/PAA, which reinforces that *Turbo S* can release SR717 in tumors and activate the STING pathway and its downstream signals in tumor cells.

**Figure 4:**
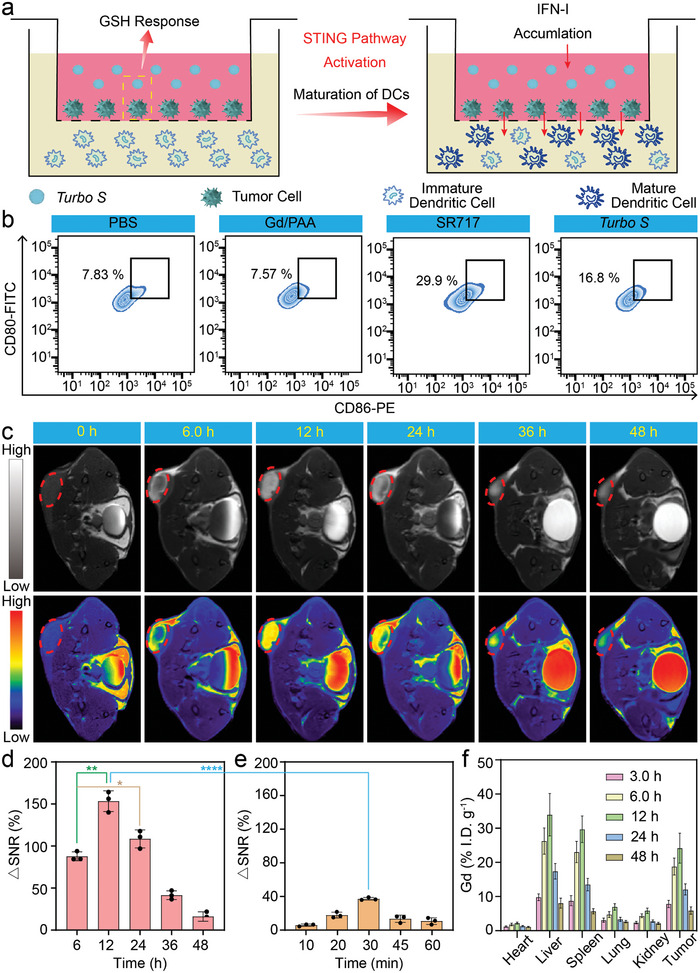
Maturation of DCs, MRI performance and biodistribution of *Turbo S* in vivo. a): Schematic illustration of the transwell plates for analysis of STING pathway activation and DCs maturation. b): The maturity of DCs after 24 h of treatment with PBS, Gd/PAA (*C*_Gd_ = 0.625 mm), free SR717 (*C*_SR717_ = 0.50 mm), or *Turbo S* (*C*_Gd_ = 0.625 mm, *C*_SR717_ = 0.50 mm) determined by flow cytometry (CD11c^+^‐APC, CD80^+^‐FITC, CD86^+^‐PE). c): *T*_1_‐weighted grayscale images and the corresponding pseudo‐color images of 4T1 tumor‐bearing mice at different time points after intravenous injection of *Turbo S* (*C*_Gd_ = 5.0 mg kg^−1^) observed on a 3.0 T clinical MRI scanner. d,e): Quantitative analysis of ΔSNR from *T*_1_‐weighted MR images at different time points for *Turbo S* (d), or Gadovist (e). Mean ± SD, *n* = 3. ^*^ *p* < 0.05, ^**^ *p* < 0.01, ^****^ *p* < 0.0001. f): Biodistribution of Gd in major organs (heart, liver, spleen, lung, kidney) and tumors at different time points after *i.v*. injection of *Turbo S* (*C*_Gd_ = 5.0 mg kg^−1^).

To further assess the biocompatibility of *Turbo S*, the cell viability was quantitatively assessed by the methyl thiazolyl tetrazolium (MTT) assay. As depicted in Figure S20 (Supporting Information), Gd/PAA exhibits almost no cytotoxicity on 4T1, MC38, or human normal hepatocytes L02 cells. Similar results are also observed for *Turbo S* (Figure S21, Supporting Information). That's because the cytotoxic effect of STING pathway on tumors is mediated by immune cells. When the STING pathway of tumor cells is activated, the downstream signals such as TBK1 and IRF3 are sequentially phosphorylated, and P‐IRF3 enters the nucleus to produce IFN‐I. IFN‐I can recruit and promote the activation of DCs, improve the expression of major histocompatibility complex (MHC)‐II and costimulatory factors CD80 and CD86 on the cell surface, thereby enhancing the ability of DCs antigen presentation. Mature DCs highly express CD80 and CD86 co‐stimulatory molecules on their surface, which is conducive to the activation of T cells.^[^ ^22^ ^]^ As illustrated in **Figure** **4**a, transwell plates were employed to evaluate the maturation of DCs, in which dendritic cells (DC 2.4) and 4T1 cells were incubated with PBS, Gd/PAA, free SR717, or *Turbo S*. The DCs were stained with CD11c‐APC, CD80‐FITC, and CD86‐PE, and the flow cytometry was applied to evaluate the maturation and differentiation of DCs. As shown in Figure 4b, it is evident that the maturation levels of DCs in the free SR717 and *Turbo S* groups reach 29.9% and 16.8% respectively, significantly exceeding those in the group of PBS (7.83%) or Gd/PAA (7.57%). The higher DC maturation in the group of free SR717 than the group of *Turbo S* can be ascribed to the faster cellular uptake of small molecule SR717 than *Turbo S*. The maturation of DCs driven by *Turbo S* potentially enables MRI‐guided activation of the STING pathway for immunotherapy in vivo.

### 2.4 Validation and Evaluation of the Performance of *Turbo S* In Vivo

To further explore the MRI performance of *Turbo S* in vivo, the half‐life of *Turbo S* in the bloodstream was measured by ICP‐OES to predict the approximate time window for MRI of tumors. Figure S22 (Supporting Information) reveals that the blood half‐life (t_1_/_2_) of *Turbo S* is 5.52 h, and the concentration of Gd in the blood steadily decreases over time. Figure 4c and Figure S23 (Supporting Information) show the *T*_1_‐weighted MR images (3.0 T) of 4T1 tumor‐bearing mice pre‐ or post‐injection (*i.v*.) of *Turbo S* and Gadovist, respectively. From the MRI images of mice injected with *Turbo S*, a pronounced intensification of the MRI signal can be observed at the tumor site with the peak at 12 h. Notably, the MRI signal intensity of tumors at 24 h post‐injection persists at a higher level than that at 6.0 h, indicating sustained intensification of the MRI signal by our *Turbo S*. This can be attributed to the significantly longer half‐life (5.52 h) of *Turbo S* than that of Gd/PAA (15 min). Additionally, the gradual release of Gd/PAA from *Turbo S* specifically within the tumor site significantly enhances the MRI efficacy, thereby enabling the sustained MRI visualization of tumors.^[^ ^19^ ^]^ In addition, the Gadovist with a small molecular weight (Mw = 547.57) undergoes rapid renal excretion, resulting in the strongest *T*_1_ signal of tumors at 30 min after intravenous administration (Figure S23, Supporting Information). Subsequently, the signal‐to‐noise ratios (SNR) of tumors at various time points post‐injection of *Turbo S* and Gadovist are calculated by the following Equations (3) and (4):

(3) $$\mathrm{SNR}=SI_{\mathrm{mean}}/SD_{\mathrm{noise}}$$

(4) $$\Delta \mathrm{SNR}=\left(\mathrm{SN}R_{\mathrm{post}}-\mathrm{SN}R_{\mathrm{pre}}\right)/\mathrm{SN}R_{\mathrm{pre}}\times 100\%$$

Figure 4d,e depicts the ΔSNR values for *Turbo S* and Gadovist. *Turbo S* provides a sustained strong *T*_1_‐MRI signal of tumors with higher ΔSNR values than that of Gadovist (36.14 ± 1.61%) (^****^ *p* < 0.0001). Additionally, the ΔSNR value of *Turbo S* at 12 h (153.49 ± 12.33%) or 24 h (111.17 ± 6.48%) is also significantly higher than that at 6.0 h (87.69 ± 5.33%) (^**^ *p* < 0.0011, ^*^ *p* < 0.05), which indicates that *Turbo S* exhibits exceptionally higher *T*_1_‐MRI efficacy for tumors than Gadovist. Compared with Gd/PAA and Gadovist, *Turbo S* can achieve *T*_1_‐MRI signal re‐enhancement for tumors through its extended half‐life and specific release of Gd/PAA, significantly prolonging the imaging time.

Figure 4f shows the bio‐distribution of Gd in major organs (heart, liver, spleen, lung, kidney) and tumors measured by ICP‐OES at 3.0, 6.0, 12, 24, and 48 h following the administration of *Turbo S*. A continuous increase of Gd content is observed in various tissues within the first 12 h, followed by a rapid decline after 12 h, indicating the ongoing clearance of *Turbo S* from organs. The liver and spleen are the two major organs that accumulate these nanoparticles because the reticuloendothelial system of the liver and spleen captures nanoparticles.^[^ ^23^ ^]^ Based on the enhanced permeation and retention (EPR) effect, nanoparticles can passively target the tumor tissues.^[^ ^24^ ^]^ Therefore, the distribution of *Turbo S* in normal organs changes over time similarly to that in tumor tissues. In addition, because the particle size of *Turbo S* is much larger than 6.0 nm, the *Turbo S* is relatively less enriched in the kidneys compared to the livers, spleens, and tumors.

After being released from *Turbo S*, Gd/PAA chelates can be transported to the kidney and bladder, and finally be excreted via urine, rather than remaining in the body for a prolonged period of time. Additionally, the accumulation of Gd in tissues reached its peak at 12 h after *i.v*. injection, which aligns with the results obtained from the 3.0 T MRI of tumor‐bearing mice. That's because our *Turbo S* has a long half‐life in the blood and can accurately release Gd/PAA in tumors. Overall, the *Turbo S* demonstrates exceptional MRI capabilities in vivo with remarkable contrast enhancement and sustained imaging efficacy, indicating potential for future clinical application as an MRI contrast agent.

### 2.5 Evaluation for the Antitumor Efficacy of *Turbo S* In Vivo

**Figure 5:**
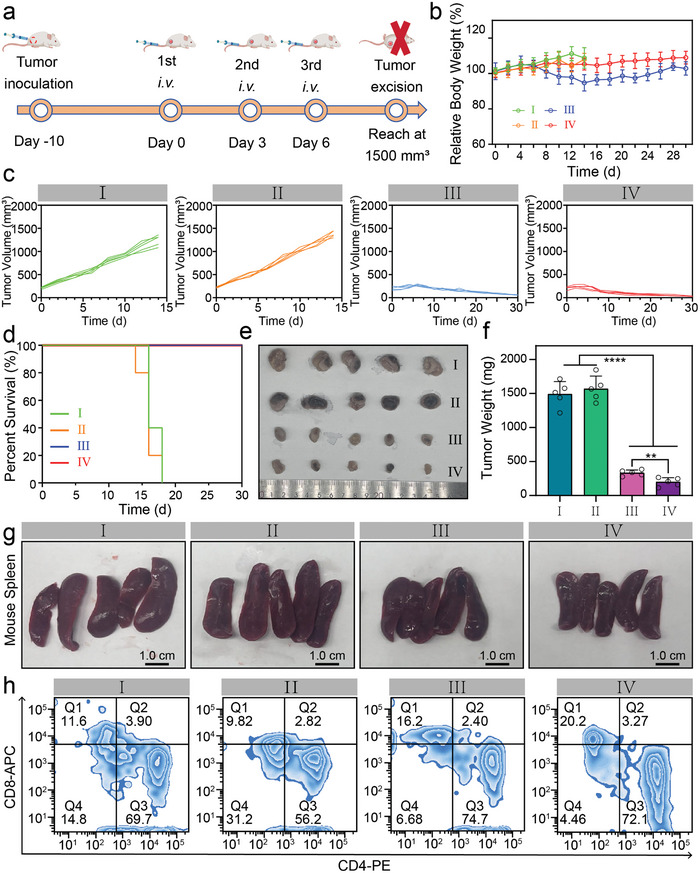
Anti‐tumor efficacy of *Turbo S* in vivo. a): Schematic illustration for the establishment of 4T1 tumor‐bearing mice model and the following treatment schedule. b): Relative body weight of tumor‐bearing mice after *i.v*. injection of saline (I), Gd/PAA (II, Gd: 5.0 mg kg^−1^), free SR717 (III, SR717: 30 mg kg^−1^), or *Turbo S* (IV, Gd: 5.0 mg kg^−1^, SR717: 8.9 mg kg^−1^). c): Tumor volumes of group I–IV. d): Survival rates of the tumor‐bearing mice of the group I‐IV. e,f): Tumor photos (e), and tumor weights (f) for the above‐mentioned group I–IV. Mean ± SD, *n* = 5. ^**^ *p* < 0.01, ^****^ *p* < 0.0001. g): Spleen photos of the tumor‐bearing mice in the group I–IV. h): The percentage of CD4^+^ T cells (Q3, CD3e^+^‐FTIC, CD4^+^‐PE, CD45^+^‐PE‐Cy5) and CD8^+^ T cells (Q1, CD3e^+^‐FITC, CD8a^+^‐APC, CD45^+^‐PE‐Cy5) in the spleen tissues of the tumor‐bearing mice in the group I–IV, measured by flow cytometry.

The in vivo antitumor effectiveness of diverse therapeutic interventions was subsequently evaluated in 4T1 tumor‐being mice. **Figure** **5**a presents the schematic illustration for the procedure of immunotherapy on 4T1‐bearing mice. The relative body weight of mice for the group I–IV is shown in Figure 5b. It can be observed that the body weight of mice in the groups of saline (I) and Gd/PAA (II) generally increase over time. However, in the group III (SR717 dosage: 30 mg kg^−1^), a decreasing trend of the mouse weight is observed, indicating systemic toxicity of free SR717. In the group IV (Gd: 5.0 mg kg^−1^; SR717: 8.9 mg kg^−1^), and the body weight of mice remains relatively stable, suggesting a good biocompatibility of *Turbo S* in vivo. The tumor growth profiles (Figure 5c) indicate that the anti‐tumor effects of the saline and Gd/PAA groups were negligible. Tumor growth curves of the group IV (*Turbo S*) with only 8.9 mg kg^−1^ of SR717 dosage show even better tumor inhibition than that of the group III with 30 mg kg^−1^ of SR717 dosage. These results demonstrate that our *Turbo S* has a much better tumor therapeutic efficacy and lower side effect than free SR717. The mice survival curves of each group are shown in Figure 5d. Tumors in the group of saline or Gd/PAA are not effectively inhibited, resulting in the euthanasia of mice between days 14 and 18. However, mice in the groups of free SR717 and *Turbo S* show higher survival rates than group of saline or Gd/PAA due to the high tumor therapeutic efficacy.

The tumor photos from each group and their corresponding weights are shown in Figure 5e,f, respectively. It is evident that the average tumor size of mice in the group *Turbo S* proved to be the smallest, followed by the group of free SR717, while mice in the group of Gd/PAA or saline gain large tumor masses. This is owing to a longer half‐life of *Turbo S*, which causes a sufficient amount of SR717 to be released in the TME through the EPR effect even at lower administration concentrations, triggering a strong immune response for tumor treatment. *Turbo S* at low dosage (8.9 mg kg^−1^ of SR717) can surpass the therapeutic efficacy of free SR717 at high dosage (30 mg kg^−1^), which indicates the turbocharging characteristics in vivo, achieving an enhanced therapeutic efficacy.

We further evaluated the anti‐tumor immune response triggered by *Turbo S* in vivo. It is well known that the spleen is a crucial immune organ in the body, regulating immune response and maintaining immune balance and stability. As shown in Figure 5g and Figures S24 and S25 (Supporting Information), it is also evident that the spleens of mice in the groups free SR717 and *Turbo S* are significantly smaller compared with the groups of saline and Gd/PAA. That's because the presence of tumors stimulates the immune system of mouse, leads to an increase of immune cells in the spleen, and consequently causes splenomegaly. As the treatment progresses, tumors in the groups of free SR717 and *Turbo S* shrink, while tumors in the other groups continue to grow. The continuous enlargement of tumor tissues leads to hypersplenism in mice, further enlarging the volume of the spleen.^[^ ^25^ ^]^ The spleens of the groups treated with free SR717 and *Turbo S* are still larger than those of healthy mice, due to the fact that small tumors are still present in the mice from these two groups, and the spleen tissue continues to express various immune cells, thereby maintaining a larger volume than in healthy mice. The smallest spleen in the group of *Turbo S* is mainly attributed to the activation of the STING pathway by SR717 released in the TME.

CD4^+^ T cells possess the ability to regulate or assist other T cells in participating in immune responses, playing a crucial role in orchestrating the overall immune reaction. Meanwhile, CD8^+^ T cells have the capacity to directly kill tumor cells, exerting a potent cytotoxic effect against them. The expression levels of CD8^+^ T cells in the spleens in each group were assessed by flow cytometry. The ratio of CD8^+^ T and CD4^+^ T cells in the group of Gd/PAA was the lowest, indicating that Gd/PAA has almost no effect on immunotherapy in the body. The proportion of CD8^+^ T cells in the group of *Turbo S* was significantly higher than the other three groups (^**^ *p* < 0.01), while the proportion of CD4^+^ T cells showed minor change compared to the groups of saline and free SR717 (Figure 5h; Figure S26, Supporting Information). These results demonstrate that the immune response induced by our *Turbo S* is primarily mediated by CD8^+^ T cells, which can recruit a substantial number of T cells to eliminate tumor cells and elicit a stronger immune response.

**Figure 6:**
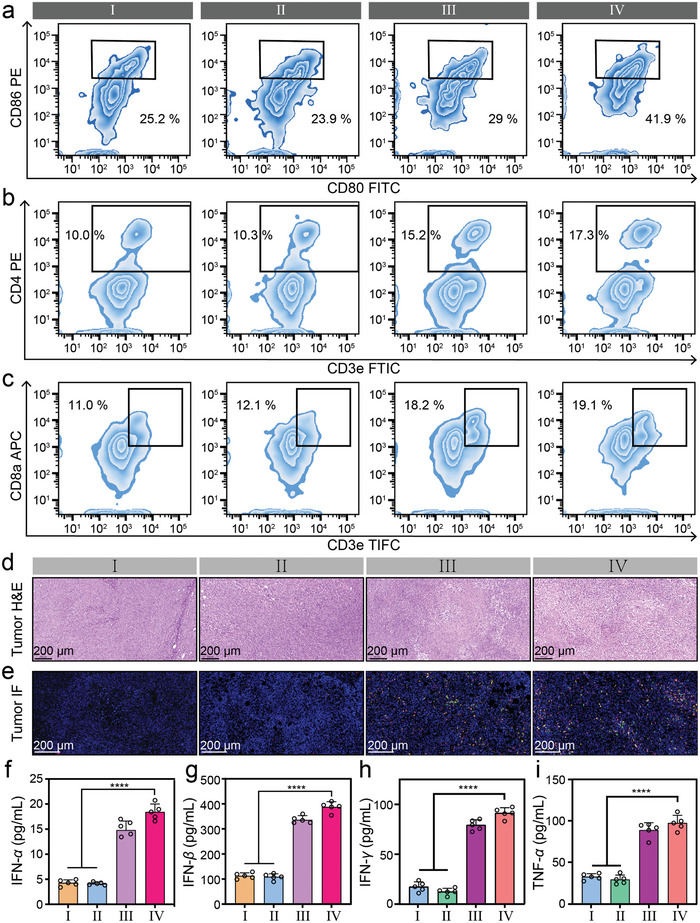
Expression of immune cells and related cytokines after immune activation. a): Expression of DCs (CD11c^+^‐APC, CD80^+^‐FITC, CD86^+^‐PE) in lymph nodes of tumor‐bearing mice after *i.v*. injection of saline (I), Gd/PAA (II, Gd: 5.0 mg kg^−1^), free SR717 (III, SR717: 30 mg kg^−1^), or *Turbo S* (IV, Gd: 5.0 mg kg^−1^, SR717: 8.9 mg kg^−1^). b,c): Expression of CD4^+^ T cells (CD3e^+^‐FTIC, CD4^+^‐PE, CD45^+^‐PE‐Cy5) (b), and CD8^+^ T cells (CD3e^+^‐FITC, CD8a^+^‐APC, CD45^+^‐PE‐Cy5) (c) in the tumor tissues of the group I–IV. d,e): Representative optical microscope images of tumor tissue sections stained with H&E (d), or immunofluorescence (e) after treatments of the group I (saline), II (Gd/PAA), III (free SR717), or IV (*Turbo S*). Blue: DAPI for cell nuclei; red: Cy3 for CD3^+^ T cells; green: FITC for CD4^+^ T cells; yellow: Cy5 for CD8^+^ T cells. f–i): The expression levels of cytokines IFN‐α (f), IFN‐β (g), IFN‐γ (h), and TNF‐α (i) of the group I–IV detected by ELISA Kits. Mean ± SD, *n* = 5. ^****^ *p* < 0.0001.

Mature DCs possess a unique capability to present tumor‐associated antigens to T lymphocytes, effectively acting as sentinels that ignite a tailored, tumor‐specific adaptive immune response. By undergoing maturation and differentiation, the DCs transform into potent activators of the immune system, orchestrating the recruitment and activation of effector T cells that are specifically geared toward recognizing and eliminating cancer cells. Therefore, a meticulous evaluation was conducted on the maturation and differentiation processes of DCs within the tumor‐draining lymph nodes (TDLNs) of each group of mice. **Figures** **6**a and S27 (Supporting Information) show the maturation of DCs in the lymph nodes of mice in each group. The group of *Turbo S* exhibits a much higher level of DCs maturation than other treatment groups (^**^ *p* < 0.01).

Mature DCs migrate to the tumor site through lymphatic vessels and blood circulation. Furthermore, upon evaluating the maturation and differentiation of DC cells in the tumors on various groups of mice, a significant increase in the degree of maturation of DC cells was observed in the group of *Turbo S* (Figure S28, 29a, Supporting Information, (^**^ *p* < 0.01). These mature DC cells that migrate to the tumor site present the processed antigen peptide‐MHC molecular complexes to tumor‐infiltrating T cells. Subsequently, the expression of T cells within the tumor was also evaluated. *Turbo S* exhibits the highest activation levels of CD4^+^ T cells and CD8^+^ T cells in tumor tissues (Figure 6b,c; Figure S29b,c, Supporting Information). The expression of CD4^+^ T cells arises from the highly immunogenic cancer cell fragments generated after cancer cells are attacked by CD8^+^ T cells, which subsequently trigger immunogenic cell death (ICD). These tumor cell fragments are then taken up by antigen‐presenting cells (APCs) and cross‐presented to both CD4^+^ and CD8^+^ T cells, leading to the activation and proliferation of CD4^+^ T cells.^[^ ^26^ ^]^ Compared with the group of free SR717 (30 mg kg^−1^), the group of *Turbo S* with 3.4 times lower SR717 dosage has a stronger immune activation because of the high delivery effect. *Turbo S* can travel through the bloodstream, gradually releasing SR717 precisely within the TME for more efficient and targeted delivery. This phenomenon is similar to the power boost from turbo lag in an automotive system. Overall, our *Turbo S* can foster a comprehensive activation of the STING pathway, thereby amplifying the efficacy of tumor immunotherapy and holding great promise for advancing cancer treatment strategies.

The tumors of each treated group were examined by hematoxylin and eosin (H&E) staining (Figure 6d). Significant damage of tumor cells can be observed in the groups of free SR717 and *Turbo S*, rather than the groups of saline and Gd/PAA. Immunofluorescence sectioning results (Figure 6e; Figure S30, Supporting Information) provide compelling evidence that *Turbo S* can induce robust tumor‐specific T cell and NK immunity. Specifically, a marked increase in the infiltration of CD4^+^ T, CD8^+^ T, and NK cells is observed within the tumors of mice treated with *Turbo S*, underscoring the effectiveness of *Turbo* S in promoting T cell recruitment.

Stimulation of the STING pathway in cancerous cells triggers the release of IFN‐I, a crucial factor that accelerates the development of mature dendritic cells (DCs) and elevates the presence of CD8^+^ T cells. This process stimulates CD8^+^ T cells to secrete IFN‐γ, ultimately triggering antitumor immune responses. The binding of IFN‐I to the IFN‐αR receptor initiates a downstream signaling cascade, activating Janus kinase 1 (JAK1) and tyrosine kinase 2 (TYK2). These kinases then phosphorylate signal transducing activators of transcription (STAT) 1 and STAT2, enhancing the production of diverse proinflammatory cytokines, TNF‐α being one of them, which further strengthens the immune system's attack on tumor cells. Therefore, the levels of cytokines (IFN‐α, IFN‐β, IFN‐γ, and TNF‐α) in the blood from each group were measured by ELISA Kits (Figure 6f–i), and the standard curves for IFN‐α, IFN‐β, IFN‐γ, and TNF‐α are shown in Figure S31a–d (Supporting Information), respectively. It is obvious that the expression levels of these cytokines in the mice after treatment with free SR717 or *Turbo S* are significantly higher than that of saline and Gd/PAA. IFN‐γ has been shown to promote the polarization of M2 toward M1 macrophages.^[^ ^27^ ^]^ M1 macrophages can secrete certain cytokines, such as TNF‐α, which can activate CD4^+^ T cells and promote their proliferation and differentiation. Immunofluorescence sectioning results (Figure S32, Supporting Information) demonstrate that *Turbo S* enhances the intratumoral abundance of M1 macrophages while significantly reducing the expression of M2 macrophages within the tumors.

Concurrently, the intratumoral abundance of immune suppressor cells, myeloid‐derived suppressor cells (MDSCs), and regulative T cells (Treg), is also significantly downregulated (Figures S33 and S34, Supporting Information). This is due to the STING pathway triggered by SR717 released from *Turbo S*, which effectively inhibits the expression of Treg and MDSC through various mechanisms including activating immune responses, remodeling the TME, inhibiting the expansion and activation of MDSCs, and promoting the reprogramming of MDSCs.

Next, the antitumor effects of *Turbo S* in mice bearing other types of tumor cells are investigated. Free SR717 still exhibits a certain degree of cytotoxicity in CT26 and MC38 tumor‐bearing mice, resulting in a moderate reduction in body weight (Figures S35a and S36a, Supporting Information). The tumor therapeutic effects in the groups of free SR717 and *Turbo S* are significantly better than those in the groups of saline and Gd/PAA (Figures S35b and S36b, Supporting Information). Additionally, the group of free SR717 shows signs of recurrence in the MC38 model, whereas *Turbo S* demonstrates robust therapeutic effects and survival rates across all three models, including 4T1, CT26, and MC38 (Figure 5d; Figures S35c and S36c, Supporting Information).

A 4T1 tumor re‐challenge experiment is conducted after the treatment of three different subcutaneous tumor models (Figure S37a, Supporting Information). Compared with the group of saline, the re‐challenge tumor growth in the group of *Turbo S* is slow and nearly cured. Although the re‐challenge tumor growth in the group of free SR717 is also relatively slow, its therapeutic efficacy is still inferior to that of the *Turbo S* group (Figure S37b, Supporting Information). In addition, *Turbo S* still maintains a high survival rate in the re‐challenge experiment (Figure S37c, Supporting Information), which further demonstrates the good efficacy of *Turbo S* for tumor immunotherapy.

Cancer cells readily infiltrate various organs and tissues within the body, with lung tissue being a particular vulnerability. Notably, 4T1 cells represent aggressive breast cancer cells characterized by their proficiency in establishing remote metastases, notably targeting the lungs. Immunotherapy has been proven to be effective in the treatment of metastatic breast cancer. To evaluate the anti‐metastatic ability of *Turbo S*, the lungs of the mice after various treatments were acquired on the twelfth day and observed after soaking in Bouin's solution. As shown in Figure S38 (Supporting Information), a stark contrast emerges in the extent of tumor metastases on the lung surface among different treatment groups. Specifically, the lungs of mice treated with saline (group I) and Gd/PAA (group II) exhibit a substantial area of metastatic tumors, underscoring the limited efficacy of these treatments in controlling metastasis. However, the mice treated with either free SR717 (group III) or *Turbo S* (group IV) display a remarkable reduction of tumor metastases in the lungs, which demonstrates the potent anti‐metastatic capabilities of *Turbo S* at low SR717 dosage and reinforces the therapeutic potential of *Turbo S* as an innovative immunotherapy.

### 2.6 In Vivo Biosafety of *Turbo S*

**Figure 7:**
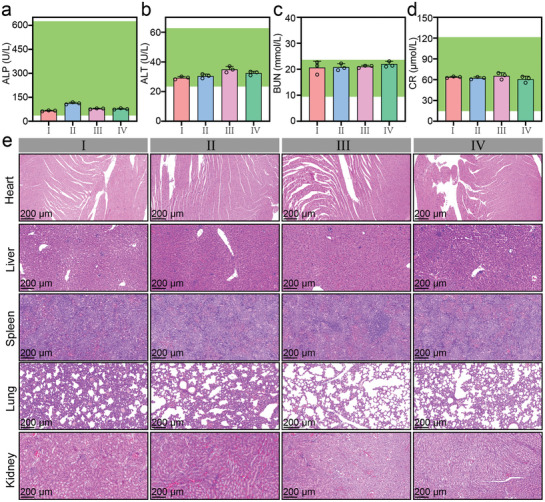
Biosafety evaluation of *Turbo S*. a–d): Liver function markers of alanine aminotransferase (ALP) (a), and alkaline phosphatase (ALT) (b), and kidney function markers of blood urea nitrogen (BUN) (c), and creatinine (CR) (d) for tumor‐bearing mice in the saline (I), Gd/PAA (II, Gd: 5.0 mg kg^−1^), free SR717 (III, SR717: 30 mg kg^−1^), or *Turbo S* (IV, Gd: 5.0 mg kg^−1^, SR717: 8.9 mg kg^−1^). The range of blue to red dotted lines indicates the normal range for each parameter. e): Representative optical microscopic images of H&E‐stained major organs (heart, liver, spleen, lung, and kidney) for the tumor‐bearing mice after 7.0 days of treatment in the group I–IV. Mean ± SD, *n* = 3.

The hemolysis ratio of *Turbo S* is less than 2.0% even at a concentration of 500 µg mL^−1^, suggesting the good blood compatibility of *Turbo S* (Figure S39, Supporting Information). Furthermore, the routine blood indices, e.g., white blood cell count (WBC), red blood cell count (RBC), hematocrit (HCT), hemoglobin (HGB), mean corpuscular hemoglobin (MCH), hemoglobin concentration (MCHC), coefficient of variation of red blood cell distribution width (RDW_CV), platelet distribution width (PDW), lymphocyte percentage (Lym), median cell percentage (Mid), granulocyte (GR), and plateletocrit (PCT), remain within normal ranges after treatment with *Turbo S* (Figure S40, Supporting Information). Similarly, serum biochemistry markers such as alanine transaminase (ALT), alkaline phosphatase (ALP), creatinine (CR), and blood urea nitrogen (BUN) were unaffected by *Turbo S* treatment, indicating normal liver and kidney function (**Figure** **7**a–d). Notably, the treated mice exhibited no significant inflammation or infection, highlighting low immunogenicity for the *Turbo S*.

The MTT results (Figure S21, Supporting Information) show that *Turbo S* with low dose of SR717 has good biosafety on tumor cells (4T1, MC38) and L02 cells. The CT26 or MC38 tumor‐bearing mice after *i.v*. injection of *Turbo S* maintains good weight changes (Figures S35a and S36a, Supporting Information), which can be ascribed to the low dose of SR717 loaded in *Turbo S* and the fact that it took some time to release SR717.

Gd^3+^‐induced nephrogenic fibrosis and brain deposition have been warned by the FDA. However, in our Gd/PAA chelate, Gd^3+^ exhibits strong coordination with carboxyl groups. The Gd/PAA chelate is a hydrophilic contrast agent, and it's difficult to cross the blood‐brain barrier (BBB) due to its large molecular weight (3927 Dalton).

The microscopic examination of H&E‐stained organs (heart, liver, spldeen, lung, and kidney) from tumor‐bearing mice after 7.0 or 30 days of treatment by *Turbo S* reveals no inflammatory lesions or tissue damage (Figure 7e; Figure S41, Supporting Information), which reinforces the biosafety of *Turbo S*. The negligible toxicity of *Turbo S* to normal tissues, including negligible long‐term toxicity (30 days), is attributed to its high accumulation in tumors with negligible release of Gd/PAA and SR717 under normal physiological conditions.

## 3 Conclusion

In summary, to alleviate the disadvantages of STING agonists (poor stability, low delivery efficiency, and potential toxicity) and reduce the potential renal fibrosis and brain deposition caused by GBCAs, a Turbo‐charging system‐like GBCA, termed as *Turbo S*, was designed and constructed for MRI‐guided STING pathway‐activated cancer immunotherapy. Typically, Gd/PAA macrochelate and SR717 were conjugated to the two terminal amino groups of CA, and the self‐assembly of SR717‐CA@Gd/PAA macromolecules resulted in the generation of SR717‐CA@Gd/PAA self‐assembled nanoparticles (SAN), which is termed as *Turbo S* because of its similarity with the Turbo‐charging system of cars. In vivo experimental results show that *Turbo S*, as a novel *T*_1_‐weighted MRI contrast agent, significantly prolongs the duration of MRI signals at the tumor site, facilitating precise monitoring of therapeutic responses. Because *Turbo S* can traverse biological barriers and selectively release SR717 within the TME, *Turbo S* with a low dosage of SR717 (8.9 mg kg^−1^) can surpass the therapeutic efficacy of free SR717 with a high dosage (30 mg kg^−1^). Both subcutaneous tumor and lung metastasis experiments demonstrate the robust antitumor and anti‐metastatic effects of *Turbo S*. Furthermore, *Turbo S* exhibits superior biosafety. The timed release of Gd/PAA and SR717 from *Turbo S*, combined with enhanced MRI performance and more effective innate immune activation, resembles the power boost induced by a turbocharging system in automobiles. By integrating targeted drug delivery, real‐time MRI monitoring, and potent immune activation, our *Turbo S* emerges as a highly promising candidate for cancer immunotherapy.

## 4 Experimental Section

### Statistics Analysis

Statistics Analysis was performed by GraphPad Prism 8.0 software (GraphPad, Inc. La Jolla, CA, USA), and quantitative data were presented as the Mean ± SD. The sample sizes (*n*) and probability (P) values for each experiment were indicated in detail in figure legends. Statistical comparisons between two groups were made using two‐tailed unpaired Student's *t*‐tests, while multiple group comparisons were assessed using One‐way ANOVA. Survival analysis was plotted using Kaplan–Meier curves. The significance level was fixed as ^*^ *p* < 0.05, ^**^ *p* < 0.01, ^***^ *p* < 0.001, or ^****^ *p* < 0.0001.

### Ethic Approval

All animal procedures were performed in accordance with the guidelines for the Care and Use of Laboratory Animals of Southern Medical University, and approved by the Animal Ethics Committee of Southern Medical University. The assigned approval/accreditation number is SYXK (YUE) 2021‐0167.

## Conflict of Interest

The authors declare no conflict of interest.

## Author Contributions

B.R., G.L., Z.S., and S.N. conceived and designed the project. B.R. and S.Y. finished most of the experiments, analyzed most of the data, and wrote the original draft. Z.L. and Y.H. helped with the synthesis of Gd/PAA and SR717‐CA1‐6@Gd/PAA SAN. H.C., C.C., and F.Q. helped with the animal experiments and the immune flow cytometry experiments. J.Y., Q.F., and C.Y. acquired and analyzed the MRI data. G.W. and R.Z. helped with CLSM imaging of cells. L.H. and J.Z. helped with TEM observations. G.L., Z.S., and S.N. helped with writing review and editing. All authors discussed the results and commented on the manuscript.

## Supporting information



Supporting Information

## Data Availability

The data that support the findings of this study are available from the corresponding author upon reasonable request.
