# Supplementary material for: A Turbo‐Charging System‐Like Contrast Agent for MRI‐Guided STING Pathway‐Activated Cancer Immunotherapy
Source: Adv Sci (Weinh). 2024 Nov 3;12(1):2410432. doi: 10.1002/advs.202410432 (PMC11714149; doi:10.1002/advs.202410432)
Supplement: Supplementary file 1 — Supporting Information [file ADVS-12-2410432-s001.docx]

Supporting Information

**A Turbo-Charging System-Like Contrast Agent for MRI-Guided STING Pathway-Activated Cancer Immunotherapy**

*Bin Ren, Sihua Yan, Zongheng Li, Ya Huang, Haobin Cai, Jing Yang, Qingdeng Fan, Chunmei Chen, Fanchao Que, Guochao Wu, Lin Huang, Ruilong Zhou, Jiaoyang Zhu, Chenggong Yan, Gang Liu*, Zheyu Shen*, Shipeng Ning**

B. Ren, Z. Li, Y. Huang, H. Cai, Dr. J. Yang, Dr. Q. Fan, Dr. C. Chen, F. Que, G. Wu, L. Huang, R. Zhou, J. Zhu, Prof. Z. Shen

School of Biomedical Engineering, Southern Medical University, 1023 Shatai South Road, Guangzhou, Guangdong 510515, China.

E-mail: [sz@smu.edu.cn](mailto:sz@smu.edu.cn)

S. Yan, Prof. S. Ning

Department of Breast Surgery, The Second Affiliated Hospital of Guangxi Medical University, Nanning 530000, China.

E-mail: nspdoctor@sr.gxmu.edu.cn

Prof. C. Yan

Medical Imaging Center, Nanfang Hospital, Southern Medical University, 1023 Shatai South Road, Guangzhou, Guangdong 510515, China.

Prof. G. Liu

State Key Laboratory of Molecular Vaccinology and Molecular Diagnostics, Center for Molecular Imaging and Translational Medicine, School of Public Health, Xiamen University, Xiamen, Fujian 361102, China.

E-mail: gangliu.cmitm@xmu.edu.cn

**EXPERIMENTAL SECTION**

**Materials and reagents:** Gadolinium chloride hexahydrate (GdCl_3_·6H_2_O, 99.9 %) was provided by Macklin. N-(3-Dimethylaminopropyl)-N’-ethylcarbodiimide (EDC, ≥ 97 %), N-Hydroxy succinimide (NHS, ≥ 99 %), poly(acrylic acid sodium salt) (average Mw ~ 5100) and Rhodamine (R6G) were purchased from Sigma-aldrich (USA). Cystamine Dihydrochloride (CA, ≥ 99 %) was purchased from MedChemExpress (Shanghai, China). SR717 was purchased form Wuhan Qiongge Biotechnology Co., Ltd. 2-(4-Amidinophenyl)-6-indolecarbamidine dihydrochloride (DAPI), FITC-Phalloidin and BCA protein concentration determination Kit were purchased from Beyotime Biotechnology (Shanghai, China). Thiazolyl blue tetrazolium bromide (MTT) was purchased from Shanghai Acmec Biochemical Co., Ltd. 5,5’-Dithiobis(2-nitrobenzoic acid) (DTNB) was obtained from Shanghai Dibai Chemicals Technology Co., Ltd. (Shanghai, China). Glutathione (GSH) was purchased from Shanghai Reagent Chemical Co. (Shanghai, China). Dulbecco’s modified Eagle’s medium (DMEM), penicillin-streptomycin, fetal bovine serum (FBS), and trypsin were purchased from Invitrogen. Thiol-Trace Violet 500 was purchased from AAT Bioquest. Mouse tumor necrosis factor *α* (TNF-*α*) ELISA Kit, mice *α* Interferon (IFN-*α*), mice *β* Interferon (IFN-*β*) and mice *γ* Interferon (IFN-*γ*) ELISA Kit were purchased by D&B (Shanghai, China). Hanks balanced salt solution, TBST (tris buffered saline + Tween 20) and red blood cell lysate were purchased from Absin (Shanghai) Biotechnology Co., Ltd. Hyaluronidase, IV collagenase, DNase I, 70 μm of filter membrane and SDS PAGE loading buffer were purchased from Biosharp (Shandong, China). Anti-STING antibody, Anti-IRF3 antibody, and CD206 were purchased form Abcam (USA). Phospho-STING (Ser366) antibody was purchased from Affbiotech (USA). Phospho-IRF3 (Ser396) rabbit mAb, TBK1/NAK (D1B4) rabbit mAb, Phospho-TBK1/NAK (Ser172) (D52C2) XP rabbit mAb, GR-1, and CD86 were purchased from Cell Signal Technology (CST). FoxP3 and CD49b antibody was purchased from Servare Biotech. Inc. Rat anti-mouse CD45-PE-Cy5, hamster anti-mouse CD3e-FITC, Ms CD4-PE, rat anti-Mouse CD8a-APC, Ms CD11c-APC HL3, rabbit anti-Mouse CD80-FITC and rat anti-mouse CD86-PE were purchased form BD Bioscience (USA).

**Characterizations:** Transmission electron microscope (TEM) and scanning electron microscope (SEM) were utilized to observe the morphology and structure of the nanoparticles. X-ray photoelectron spectroscopy (XPS) was taken advantage to determine the valence states of elements for the nanoparticles. The energy dispersive X-ray spectroscopy (EDS) spectra of nanoparticles were acquired from a TEM (JEM-2100F, JEOL). The size and zeta potential of nanoparticles were measured by an instrument of dynamic light scattering (DLS) (Nano-Brook 90PlusZata, Brookhaven). UV-vis spectra were measured by a spectrophotometer (Evolution 300, Thermo Fisher). Fourier transform infrared (FT-IR) spectra were recorded by a Nicolet 6700 spectrometer (Thermo Electron Corporation, Madison, WI). The Gd contents of the nanoparticles was determined by inductively coupled plasma-optical emission spectrometer (ICP-OES, Thermo Fisher). The expression of STING-associated proteins (STING, P-STING, IRF3, P-IRF3, TBK1, P-TBK1) were determined by western blot (WB, BIO-RAD) analysis.

**Synthesis of Gd/PAA:** The PAANa (Mw 5100) solution (4.0 mg/mL, 20 mL, pH = 10) was stirred (500 rpm) at 100 ^o^C. Then, the GdCl_3_ (125 mM, 0.40 mL) solution was added into the reaction system. The reaction was continued for 180 min under magnetic stirring to obtain the Gd/PAA macrochelate. Then, the obtained Gd/PAA macrochelate were purified by membrane dialysis (Mw 3.0 kDa) against ultra-pure water for three days with water change twice a day. The purified Gd/PAA were concentrated to 4.0 mM *via* rotary evaporation for further use.

**Synthesis of SR717-CA@Gd/PAA:** 10 μL of EDC (10 μM) solution, 1.0 mL of NHS solution (8.80 mg/mL), and 1.5 mL of CA solution (2.0 mM) were added into 1.5 mL of SR717 solution (2.0 mM, dissolved in DMSO) in turn. The mixture was then stirred at room temperature for 4.0 h, generating SR717-CA.

Then, 4.0 mL of the above-mentioned SR717-CA solutions was then added into 0.14-4.57 mL of the Gd/PAA-NHS solution, and the total volume of the solution was adjusted to 10 mL with water. Then, the mixed solution was stirred at room temperature for 4.0 h. After the reaction, the obtained SR717-CA@Gd/PAA were purified by membrane dialysis (Mw 3.0 kDa) against ultra-pure water for three days with water change twice a day. The purified SR717-CA@Gd/PAA were finally concentrated *via* rotary evaporation for further use.

**Synthesis of R6G-*Turbo S*:** 4.0 mL of *Turbo S* (*C*_Gd_ = 1.0 mM) were mixed with 1.0 mL of Rhodamine 6G (10 μM) under magnetic stirring at room temperature. After 24 h, the R6G-*Turbo S* was obtained by centrifugation (12000 × g, 15 min), and washed thrice with pure water. The sample was finally dispersed in 5.0 mL of water, and kept in fridge for further use.

***In Vitro* MRI Performance:** The MRI measurements were performed with a clinical MRI scanner (3.0 T, Philips, Ingenia, NL) or a high magnetic field MRI scanner (7.0 T, Bruker, PharmaScan70/16, US). The aqueous solutions of Gd/PAA or *Turbo S* were prepared with gradient *C*_Gd_ (400, 200, 100, 50.0, 25.0, or 12.5 μM). The relaxivity values of *r*1 and *r*2 were obtained as the slopes from the linear curves of relaxation rate (1/*T*1 or 1/*T*2, s^-1^) versus the Gd concentration (mM). For *T*_1_ relaxation rates at magnetic field of 7.0 T, TR is 75.8 ms, and TE is 6.0 ms. For *T*_1_ relaxation rates at magnetic field of 3.0 T, TR is 200 ms, TE is 8.2 ms. For *T*_2_ relaxation rates at magnetic field of 7.0 T, TR is 6000 ms, and TE is 120 ms. For *T*_2_ relaxation rates at magnetic field of 3.0 T, TR is 5000 ms, and TE is 80 ms. Signal intensities were measured with Image J software. The signal-to-noise ratio (SNR) and ΔSNR were calculated by the formula (1) and (2).

SNR = SI_mean_ / SD_noise_ (1)

ΔSNR = (SNR_sample_ - SNR_water_) / SNR_water_ × 100 % (2)

**GSH Consumption by *Turbo S*:** To evaluate the GSH depletion capability, 5.0 mL of *Turbo S* (1.0 mg/mL) was dispersed in 5.0 mL of GSH solution (10 mM). The mixed solutions were maintained at 37 °C under magnetic stirring for 0, 6.0, 12, 24, or 48 h. The above mixtures were then centrifuged at 10000 × g for 5.0 min. The obtained supernatants were mixed with 50 μL of DTNB (10 mg/mL), and the mixtures were then incubated for 15 min. After that, the solutions were measured by UV-vis spectrophotometer (Evolution 300, Thermo Fisher) at the wavelength of 412 nm.

In addition, TEM was also employed to examine the morphological changes of *Turbo S* after reaction with or without GSH. Typically, 5.0 mL of *Turbo S* (1.0 mg/mL) was mixed with 5.0 mL of PBS (pH 7.4) solution with or without GSH (10 mM) under magnetic stirring at 37 °C. 20 μL of the mixture was collected after 0, 12, 24, 48, or 72 h of reaction, and 20 μL of PBS with same condition was supplemented. The collected samples were finally observed by TEM (JEM-2100F, JEOL).

***In Vitro* Release Behaviors of Gd/PAA or SR717 from *Turbo S*:** 1.0 mL of *Turbo S* (*C*_Gd_ = 2.0 mM, *C*_SR717_ *=* 1.6 mM) was transferred into a dialysis bag. The dialysis bag was immerged in 200 mL of PBS at pH 7.4 with GSH (5.0, or 10 mM) in a glass bottle, which was placed in a 37 ℃ of incubator with shaking at 100 rpm. At predetermined time points (0, 2.0, 4.0, 6.0, 12, 24, 36, 48, or 72 h), 1.0 mL of the solution was taken out, and 1.0 mL of PBS with same condition was supplemented. The release of Gd/PAA was further tested by ICP-OES (ICP-OES, Thermo Fisher), and the release of SR717 was determined by UV-vis spectrophotometer (Evolution 300, Thermo Fisher).

**The Changes in MRI relaxivities after GSH Response:** 15 mL of *Turbo S* (*C*_Gd_ = 1.0 mg/mL) was mixed with 15 mL of PBS at pH 7.4 with or without GSH (20 mM) under magnetic stirring at 37 °C. 400 μL of the mixture was then collected after 0, 12, 24, 48, or 72 h of reaction. The collected samples were used to prepare solutions with gradient *C*_Gd_ (400, 200, 100, 50.0, 25.0, or 12.5 μM) for measurements on a 7.0 T MRI scanner (Bruker, PharmaScan70/16, US). The *r*1 and *r*2 values were obtained as the slopes from the linear curves of relaxation rate (1/*T*1 or 1/*T*2, s^-1^) versus the Gd concentration (mM). For *T*_1_ relaxation rates at magnetic field of 7.0 T, TR is 75.8 ms, and TE is 6.0 ms. For *T*_2_ relaxation rates at magnetic field of 7.0 T, TR is 6000 ms, and TE is 120 ms.

**Cell Culture:** mouse breast cancer cell line 4T1, mouse colon cancer cell line MC38, mouse colon cancer cells CT26, human normal hepatocyte line L02, and dendritic cells (DC 2.4) were cultured in DMEM medium supplemented with 10 % fetal bovine serum (FBS) and 1.0 % penicillin/streptomycin. The cells were incubated at 37 °C in a humidified atmosphere containing 5.0 % of CO_2_.

**Cellular Uptake:** For confocal laser scanning microscope (CLSM) analysis, 4T1 cells or MC38 cells (1.0 × 10^5^ cells) were seeded into a confocal dish, and incubated overnight to achieve adherence. The growth medium was then replaced with a fresh one (2.0 mL, without FBS) containing R6G-*Turbo S* (*C*_Gd_ = 0.50 mM). After 4.0 h of incubation, the treated cells were washed with PBS, fixed with 4.0 % of paraformaldehyde for 30 min, permeabilized with 0.10 % of Triton X-100 for 5.0 min, and blocked with 1.0 % of BSA for 30 min. The cells were then stained with FITC-Phalloidin or DAPI for 15 or 30 min, respectively. Finally, the cells were observed by CLSM (Nikon ECLIPSE Ti2).

For flow cytometry analysis: 4T1 cells or MC38 cells were seeded into 6-well plates (5.0 × 10^5^ cells/well), and incubated overnight to achieve adherence. Then, the culture medium was replaced with DMEM medium (3.0 mL) containing R6G-*Turbo S* (*C*_Gd_ = 0.50 mM). After incubation for further 4.0 h, the cells were washed twice with PBS, and harvested by trypsinization and centrifugation (800 × g, 3.0 min). The obtained cells were re-suspended in 200 μL of PBS, and then analyzed by flow cytometry (BD LSRFortessa X-20).

The cellular uptake of *Turbo S* was also quantitatively determined by ICP-OES. Briefly, 3.0 mL of 4T1, MC38 cells in complete DMEM medium were seeded in 6-well plates at a density of 2.0 × 10^5^ cells per well, and incubated at 37 ℃ for 24 h. The growth medium was then replaced with a fresh one (3.0 mL, without FBS) containing *Turbo S* (*C*_Gd_ = 0.50 mM). After incubation for 0.50, 1.0, 2.0, or 4.0 h, the cells were washed twice with PBS, treated with trypsin for 2.0 min, and then centrifuged at 800× g for 3.0 min. The obtained cells were finally digested by concentrated nitric acid for Gd measurement by ICP-OES (ICP-OES, Thermo Fisher).

**MRI Studies on Cells:** 4T1 cells in complete growth medium were seeded in 6-well plates at a density of 2.0 × 10^5^ cells per well, and incubated at 37 ℃ for 24 h to achieve adherence. The growth medium was then replaced with a fresh one (3.0 mL, without FBS) containing *Turbo S* (*C*_Gd_ = 0.50 mM). After 0, 1.0, 2.0, or 4.0 h of incubation, the cells were washed with PBS, trypsinized, centrifuged, and transferred into 1.5 mL of centrifuge tubes. 1.0 mL of agarose solution (0.80 wt %) was added to fix the cells. *T*_1_-weighted MR images were acquired by a clinical MRI scanner (3.0 T, Philips, Ingenia, NL). TE = 8.2 ms, TR = 500 ms.

**Lysosome Escape Measurements:** 4T1 cells in complete growth medium were seeded into confocal dishes at a density of 1.0 × 10^5^ cells, and allowed to adhere at 37 ℃ for 24 h. And then, the growth medium was replaced with a fresh one (1.0 mL, without FBS) containing R6G-*Turbo S* (*C*_Gd_ = 0.50 mM) for 8.0 h. After that, the treated cells were washed with PBS, fixed with 4.0 % of paraformaldehyde for 30 min, permeabilized with 0.10 % Triton X-100 for 5.0 min, and blocked with 5.0 % BSA for 30 min. The cells were then stained with DAPI and Lysotracker FITC for 30 min. Finally, the cells were observed by CLSM (Nikon ECLIPSE Ti2).

**Detection of GSH:** For confocal laser scanning microscope (CLSM) analysis, 4T1 cells were seeded into a confocal dish at a density of 1.0 × 10^5^ cells, and incubated at 37 ℃ for 24 h to achieve adherence. The growth medium was then replaced with a fresh one (2.0 mL, without FBS) with PBS, Gd/PAA (*C*_Gd_ = 0.50 mM), free SR717 (*C*_SR717_ = 0.40 mM), or *Turbo S* (*C*_Gd_ = 0.50 mM, *C*_SR717_ = 0.40 mM). After 24 h of incubation, the treated cells were washed with PBS, fixed with 4.0 % of paraformaldehyde for 30 min, permeabilized with 0.10 % of Triton X-100 for 5.0 min, and blocked with 1.0 % of BSA for 30 min. The cells were then stained with Thiol-Trace Violet, and DAPI for 30 min, respectively. Finally, the cells were observed by CLSM (Nikon ECLIPSE Ti2).

For intracellular GSH: 4T1 cells were seeded in a 6-well plate (2.0 × 10^5^ cells/well), and incubated at 37 ℃ for 24 h to achieve adherence. The culture medium was replaced with fresh one with PBS, Gd/PAA (*C*_Gd_ = 0.50 mM), free SR717 (*C*_SR717_ = 0.40 mM), or *Turbo S* (*C*_Gd_ = 0.50 mM, *C*_SR717_ = 0.40 mM). After 24 h of incubation, the cells were washed with cold PBS and collected by a cell scraper.

**Detection of STING Activation:**

For phospho-STING:

4T1 cells were seeded into a confocal dish at a density of 1.0 × 10^5^ cells, and incubated at 37 ℃ for 24 h to achieve adherence. The growth medium was then replaced with a fresh one (2.0 mL, without FBS) with PBS, Gd/PAA (*C*_Gd_ = 0.625 mM), free SR717 (*C*_SR717_ = 0.50 mM), or *Turbo S* (*C*_Gd_ = 0.625 mM, *C*_SR717_ = 0.50 mM). After 24 h of incubation, the cells were treated with ER-Tracker green at 37 ^o^C for 30 min. Then the treated cells were washed with PBS, fixed with 4.0 % of paraformaldehyde for 30 min, and blocked with 5.0 % of BSA for 30 min. The cells were then treated with phospho-STING rabbit anti-mouse primary antibody (1 : 200 in primary antibody diluent) on a shaker overnight (60 rpm, 4.0 ^o^C) and stained with goat anti-rabbit secondary antibody (red fluorescence) for 1.0 h at room temperature, and DAPI for 30 min, respectively. Finally, the cells were observed by CLSM (Nikon ECLIPSE Ti2).

For phospho-IRF3:

4T1 cells were seeded into a confocal dish at a density of 1.0 × 10^5^ cells, and incubated at 37 ℃ for 24 h to achieve adherence. The growth medium was then replaced with a fresh one (2.0 mL, without FBS) with PBS, Gd/PAA (*C*_Gd_ = 0.625 mM), free SR717 (*C*_SR717_ = 0.50 mM), or *Turbo S* (*C*_Gd_ = 0.625 mM, *C*_SR717_ = 0.50 mM). After 24 h of incubation, the treated cells were washed with PBS, fixed with 4.0 % of paraformaldehyde for 30 min, and permeabilized with 0.10 % of Triton X-100 for 5.0 min, blocked with 5.0 % of BSA for 30 min. The cells were then treated with phospho-IRF3 (1 : 200 in primary antibody diluent) rabbit anti-mouse primary antibody on a shaker overnight (60 rpm, 4.0 ^o^C) and stained with goat anti-rabbit secondary antibody (red fluorescence) for 1.0 h at room temperature, and DAPI for 30 min, respectively. Finally, the cells were observed by CLSM (Nikon ECLIPSE Ti2).

**Western Blot:** 4T1 cells were seeded in 6-well plates at a density of 2.0 × 10^5^ cells per well in complete growth medium, and allowed to adhere at 37 ℃ for 24 h. The growth medium was replaced with fresh one (2.0 mL, without FBS) containing PBS, Gd/PAA (*C*_Gd_ = 0.625 mM), free SR717 (*C*_SR717_ = 0.50 mM), or *Turbo S* (*C*_Gd_ = 0.625 mM, *C*_SR717_ = 0.50 mM). After 24 h of incubation, the cells in all dishes were washed by cold PBS. After that, the cells in each well were treated with 150 μL of RIPA lysis buffer (with PMSF) at 0 ℃ for 30 min, and the lysate was collected and centrifuged (12000 × g, 20 min, 4.0 ℃). The supernatant after centrifugation was quantified for protein concentration by a BCA assay Kit. RIPA lysis buffer and loading buffer were then added to dilute the protein of each group to a uniform concentration, followed by boiling in a metal bath (100 ℃, 10 min). SDS-PAGE gel (12 %) was taken advantage for electrophoretic separation of various protein samples. The separated protein samples on the gel were then transferred to a PVDF membrane (0.45 μm). Subsequently, the membrane was blocked with 5.0 % of BSA solution for 1.0 h. The membrane was washed three times with TBST, and incubated overnight at 4.0 ℃ with the corresponding rabbit anti-mouse primary antibody (STING, P-STING, IRF3, P-IRF3, TBK1, P-TBK1, or GAPDH) on a shaker, then washed by TBST. After that, the membrane was incubated with a goat anti-rabbit secondary antibody at room temperature for 1.0 h, then washed by TBST. Finally, the protein bands were visualized by an enhanced chemiluminescence (ECL) detection reagent in a darkroom. The expression level of each protein was quantified by Image J software and normalized to GAPDH.

**MTT Assay:** Cytotoxicities were evaluated by the methyl thiazolyl tetrazolium (MTT) assay on 4T1 cells, MC38 cells cells. Typically, all cells mentioned above were seeded in 96-well culture plates at a density of 1.0 × 10^4^ cells per well, respectively. After overnight incubation, the growth medium was replaced with a fresh one (without FBS) containing Gd/PAA or *Turbo S* with gradient *C*_Gd_ (100, 50, 25, 12.50, 6.25, 3.13, 1.56, or 0.78 μM). After 24 h of incubation, 10 μL of MTT (5.0 mg/mL in PBS) was added to each well of the 96-well plates. After 4.0 h, the culture media were replaced with 150 μL of DMSO per well to dissolve the formazan crystals. Finally, a multi-mode microplate reader (Synergy H1, BioTek Instruments, USA) was applied to measure the absorbance of each well at 490 nm.

**Maturation of DCs:** The DC 2.4 maturation was performed by a 6-well transwell system with 0.4 μm of porous membrane. The 4T1 cells were cultured in the upper wells and treated with PBS, Gd/PAA (*C*_Gd_ = 0.625 mM), free SR717 (*C*_SR717_ = 0.50 mM), or *Turbo S* (*C*_Gd_ = 0.625 mM, *C*_SR717_ = 0.50 mM) for 24 h. After replacement with fresh medium, the upper wells with 4T1 cells were transferred to other transwell plates whose bottom wells contain DC 2.4. After further 24 h of incubation, the DC 2.4 cells were stained with CD11c-APC, CD80-FITC, and CD86-PE, and then collected for the flow cytometry (BD LSRFortessa X-20) analysis to evaluate the maturation of DC 2.4.

**Tumor Model:**

Female Balb/c mice (5 weeks old, 15 ~ 20 g) were purchased from the Experimental Animal Center of Southern Medical University (Guangzhou, China). For subcutaneous tumors: the 4T1 tumor-bearing mice were prepared by subcutaneous inoculation of 4T1, CT26, or MC38 cells (2.0 × 10^6^ cells in 100 μL PBS) into the right leg of each mouse. For lung metastasis models: the 4T1 lung metastasis-bearing mice were prepared by intravenously (*i.v.*) inoculation of 4T1 cells (2.0 × 10^6^ cells in 100 μL PBS) into each mouse.

***In Vivo* Study of *T*_1_-weighted MR Imaging:** *In vivo* *T*_1_-MRI evaluation of *Turbo S* or Gadovist^®^ were performed by a 3.0 T clinical scanner (Philips, Ingenia, NL) with an 8-channel mouse coil. The parameters were shown as: TR/TE = 500/8.4 ms, FOV = 50 × 50 mm^2^, matrix size = 252 × 248, number of averages = 4, scan time = 232 s. The 4T1 tumor model was established on 4T1-beared mice. *Turbo S* (Gd dosage: 5.0 mg/kg) and Gadovist^®^ (Gd dosage: 5.0 mg/kg) were injected *i.v*. signal intensities were measured with the Image J software. The SNR and ΔSNR were calculated using the formula (3) and (4).

SNR = SI_mean_ / SD_noise_  (3)

ΔSNR = (SNR_post_ - SNR_pre_) / SNR_pre_ × 100 % (4)

***In Vivo* Pharmacokinetics:** Healthy mice were used for the pharmacokinetic analysis. Before injection of *Turbo S*, 0.1 mL of blood (as a control to eliminate the signal of Gd inside blood) was collected from each mouse, and 0.1 mL of saline was immediately injected *via* the tail vein. Subsequently, 0.1 mL of *Turbo S* (Gd dosage: 5.0 mg/kg) were *i.v.* injected *via* the tail vein. After injection, 0.1 mL of venous blood samples were collected from the orbital region at different time points (0.5, 1.0, 2.0, 4.0, 8.0, 12, 24, or 36 h), and 0.1 mL of saline was immediately supplemented *via* the tail vein. Finally, the samples were analyzed for Gd concentration by ICP-OES (ICP-OES, Thermo Fisher).

***In Vivo* Biodistribution:** When the tumor volume of 4T1 tumor-bearing mice grew up to 100-150 mm^3^, and then *i.v.* injected with 0.10 mL of *Turbo S* (Gd dosage: 5.0 mg/kg). At 3.0, 6.0, 12, 24, or 48 h post-injection, the mice were sacrificed. Subsequently, the major organs (heart, liver, spleen, lung, and kidney) and tumors were were dissected and cut into proper size. After weighting, they were digested by 5.0 mL of 68 % nitric acid, and dissolved with 10 mL of 1.0 % nitric acid solution. The Gd content of each group were determined by ICP-OES (Thermo Fisher), and expressed as a percentage of the injected Gd dose per gram of tissue.

**Subcutaneous** **Tumor Therapeutic Performance:** When the tumor volume reached to about 200-250 mm^3^, 4T1, CT26, or MC38 tumor-bearing mice were randomly divided into four groups for treatments. 100 μL of saline, Gd/PAA (Gd dosage: 5.0 mg/kg), free SR717 (SR717 dosage: 30 mg/kg), or *Turbo S* (Gd dosage: 5.0 mg/kg, SR717 dosage: 8.9 mg/kg) were *i.v.* injected into the 4T1, CT26, or MC38 tumor-bearing mice at the day 0, 3.0, or 6.0, respectively. Body weights and tumor volumes of mice in each group were recorded every other day during the treatment (the total tumor volume should be smaller than 1500 mm^3^). The tumor volume was calculated referring to the equation: Volume = (length × width × width)/2.

In another parallel groups, all the mice were sacrificed on one day that the first mouse tumor volume reached at 1500 mm^3^.The blood, tumors and major organs (heart, liver, spleen, lung, and kidney) of mice were sequentially collected. Additionally, the length and width of spleen tissues of mice were recorded, and photographs of the spleen tissues were taken. The expression of cytokines (TNF-*α*, IFN-*α*, IFN-*β*, and IFN-*γ*) in collected blood samples were tested by respective ELISA assay Kits. A portion of the tumors in each group were used for immunoflow cytometry testing, while the remaining portion was used for H&E or immunofluorescence staining.

For H&E and immunofluorescence analysis: tumor masses were weighed, photographed, fixed in 4.0 % paraformaldehyde, embedded in paraffin, sectioned, stained with hematoxylin and eosin (H&E). Immunofluorescence analysis of CD4^+^ and CD8^+^ T cells in tumor tissues was conducted, staining cell nuclei with DAPI (blue), CD3^+^ T cells with CD3 (Cy3), CD4^+^ T cells with CD4 (FITC), CD8^+^ T cells with CD8 (Cy5), M1 macrophages with CD86 (Cy3), M2 macrophages with CD206 (FITC), myeloid-derived suppressor cells (MDSC) with GR-1 (Cy3), regulatory T cells (Treg) with FoxP3 (Cy3), and natural killer (NK) cells with CD49b (FITC). Finally, the cells were observed by CaseViewer scanner (Pannoramic SCAN).

For the re-challenge studies: When the tumor volume reached to about 200-250 mm^3^, 4T1 tumor-bearing mice were randomly divided into two groups for treatments. 100 μL of free SR717 (SR717 dosage: 30 mg/kg) or *Turbo S* (Gd dosage: 5.0 mg/kg, SR717 dosage: 8.9 mg/kg) were *i.v.* injected into the 4T1 tumor-bearing mice at the day 0, 3.0, or 6.0, respectively. Meanwhile, healthy mice were *i.v.* injected with saline at day 0, 3.0, or 6.0, respectively. Then the cured 4T1 tumor-bearing mice and the healthy mice injected with saline were subcutaneously re-inoculated with 4T1 cells (2.0 × 10^6^ in 100 μL PBS). Tumor volumes of mice in each group were recorded every other day during the treatment (the total tumor volume should be smaller than 1500 mm^3^). And the tumour growth was measured until day 35. The tumor volume was calculated referring to the equation: Volume = (length × width × width)/2.

**Lung Metastasis Tumor Therapeutic Performance:** Mice were *i.v.* inoculated with 4T1 cells (2.0 × 10^6^ cells in 100 μL PBS) on day 0. 4T1 lung metastasis-bearing mice were randomly divided into four groups for treatments. 100 μL of saline, Gd/PAA (Gd dosage: 5.0 mg/kg), free SR717 (SR717 dosage: 30 mg/kg), or *Turbo S* (Gd dosage: 5.0 mg/kg, SR717 dosage: 8.9 mg/kg) were intravenously injected into the 4T1 tumor-bearing mice at the day 3.0, 5.0, or 7.0, respectively. All the mice were sacrificed on day 12, and the lungs of mice was collected and washed by PBS, then stained with Bouin’s fluid for 24 h.

**Immunity Flow Cytometry (IFC):** For the tumor tissues: the tumor tissues were cut into small pieces with scissors, and then digested with Hanks’ solution containing collagenase (0.30 g/L), hyaluronidase (0.30 g/L), and DNase (0.15 g/L). And the mixture was filtered through a 70 μm of filter membrane, washed with PBS by centrifugation (3000 × g, 5.0 min). Subsequently, the cells were stained with fluorescent antibodies (CD3-FTIC, CD4-PE, CD8-APC, CD45-PE-Cy5 for T cells; CD11c-APC, CD80-FITC, CD86-PE for DCs) and analyzed by flow cytometry (BD LSRFortessa X-20) to determine the percentages of immune cells within the tumors, including cytotoxic T cells (CTLs, CD8 T cells, CD3^+^, CD8^+^, CD45^+^), helper T cells (CD4 T cells, CD3^+^, CD4^+^, CD45^+^), and dendritic cells (CD11c^+^, CD80^+^, CD86^+^).

For the lymph nodes: the lymph nodes were ground and filtered through a 70 μm of filter membrane, and washed with PBS by centrifugation (3000 × g, 5.0 min). Subsequently, the cells were stained with fluorescent antibodies, and flow cytometry (BD LSRFortessa X-20) was utilized to analyze the percentage of mature dendritic cells in the lymphocyte populations of each group.

For spleen tissues: the spleen tissues were lysed with RBCs lysis buffer, filtered through a 70 μm of filter membrane, and washed with Hanks’ solution by centrifugation (3000 × g, 5.0 min). The cells were then stained with fluorescent antibodies and analyzed by flow cytometry (BD LSRFortessa X-20) to determine the percentages of CD4^+^ (CD3^+^, CD4^+^, CD45^+^) T cells and CD8^+^ (CD3^+^, CD8^+^, CD45^+^) T cells for assessment of the systemic immune level of splenic infiltrating T cells.

**Hemolysis Assay:** For hemolysis assay, red blood cells (RBCs) were first isolated by centrifugation of fresh blood from Balb/c mice (250 × g, 10 min). After that, the supernatant was carefully removed from the RBCs, and the lower layer of RBCs were washed with PBS until the supernatant became clear and transparent. The concentration of the collected RBCs was then diluted to 4.0 % (v/v). The *C*_Gd_ of *Turbo S* was respectively adjusted to 500, 250, 125, 62.50, 31.30, and 15.60 μg/mL by PBS. Then, 500 μL of *Turbo S* exhibiting distinct *C*_Gd_ were respectively added into 500 μL of RBCs, and the mixtures were immediately incubated in 37 ± 0.5 °C of water bath for 4.0 h. Under the same conditions, the RBCs were respectively mixed with pure water or PBS (pH = 7.4) as a positive or negative control. The samples were taken out and centrifuged (250 × g, 10 min) to remove intact red blood cells. 100 μL of the supernatants of each group were added into a 96-well plate, and the absorbance was measured at 545 nm by a microplate reader (Synergy H1, BioTek Instruments). Finally, the percentage of hemolysis was determined as (A_sample_ − A_negative control_) / (Apositive control − A_negative control_) × 100 %, where A_sample_, Apositive control, and A_negative control_ are the absorbance of the samples, the completely lysed RBCs in pure water, and zero hemolysis in PBS, respectively. Three parallel groups were tested for each concentration.

**Biosafety Evaluation of *Turbo S*:** 0.10 mL of Saline, Gd/PAA (Gd dosage: 5.0 mg/kg), SR717 (30 mg/kg), or *Turbo S* (Gd dosage: 5.0 mg/kg; SR717 dosage: 8.9 mg/kg) was *i.v.* injected into the healthy Balb/c mice. After 24 h, the blood samples of the four experimental groups mentioned previously and healthy mice were collected and centrifuged at 3000 × g for 15 min. 300 μL of the upper serum was taken out to determine the Hematology indicators, *i.e.*, alanine aminotransferase (ALT), alkaline phosphatase (ALP), blood urea nitrogen (BUN), creatinine (CR), white blood cell count (WBC), red blood cell count (RBC), hematocrit (HCT), hemoglobin (HGB), mean corpuscular hemoglobin (MCH), hemoglobin concentration (MCHC), coefficient of variation of red blood cell distribution width (RDW_CV), platelet distribution width (PDW), lymphocyte percentage (Lym), Median cell percentage (Mid), granulocyte (GR), and plateletocrit (PCT).

For the major organs toxicity detection, the mice were sacrificed at 72 h or 30 days post-injection, and the major organs (heart, liver, spleen, lung, and kidney) and tumors were collected. Then, the H&E staining method was used to observe and analyze the sections of major organs.

**Table S1**. Synthesis conditions and characterization results of the SR717-CA1-6@Gd/PAA self-assembled nanoparticles (SAN).

| Sample Nomenclature | SR717-CA/Gd Molar Ratio ^a^ | Recovery of Gd (%) ^b^ | Loading Content (%) ^c^ | Loading Efficiency (%) ^d^ | *r*_1_  [mM^-1^ s^-1^] ^e^ | *r*_2_  [mM^-1^ s^-1^] ^e^ | *r*_2_/*r*_1_ |
| --- | --- | --- | --- | --- | --- | --- | --- |
| SR717-CA1@Gd/PAA SAN | 8 | 37.3 | 2.3 | 35.3 | 11.72 | 92.23 | 7.87 |
| SR717-CA2@Gd/PAA SAN | 4 | 51.3 | 4.7 | 42.6 | 16.55 | 83.37 | 5.04 |
| SR717-CA3@Gd/PAA SAN | 2 | 73.5 | 9.7 | 59.4 | 19.28 | 77.79 | 4.03 |
| SR717-CA4@Gd/PAA SAN | 1 | 89.8 | 13.3 | 82.4 | 27.86 | 63.13 | 2.27 |
| SR717-CA5@Gd/PAA SAN | 0.5 | 96.7 | 8.6 | 92.6 | 26.47 | 71.52 | 2.70 |
| SR717-CA6@Gd/PAA SAN | 0.25 | 96.1 | 6.7 | 97.8 | 27.47 | 73.35 | 2.67 |

^a^ The *C*_Gd_ of Gd/PAA-NHS is 3.50 mM, and the concentrations of SR717-CA is 1.0 mM.

^b^ The recovery of Gd is calculated from the molar percentage of Gd in the obtained SR717-CA@Gd/PAA SAN to that in the feeding Gd/PAA-NHS.

^c^ Calculated from the mass percentage of loaded SR717 to SR717-CA1-6@Gd/PAA SAN.

^d^ Calculated from the mass percentage of loaded SR717 to the feeding SR717.

^e^ The *r*_1_ and *r*_2_ values are measured on a clinical MRI scanner system (3.0 T, Philips, Ingenia, NL).


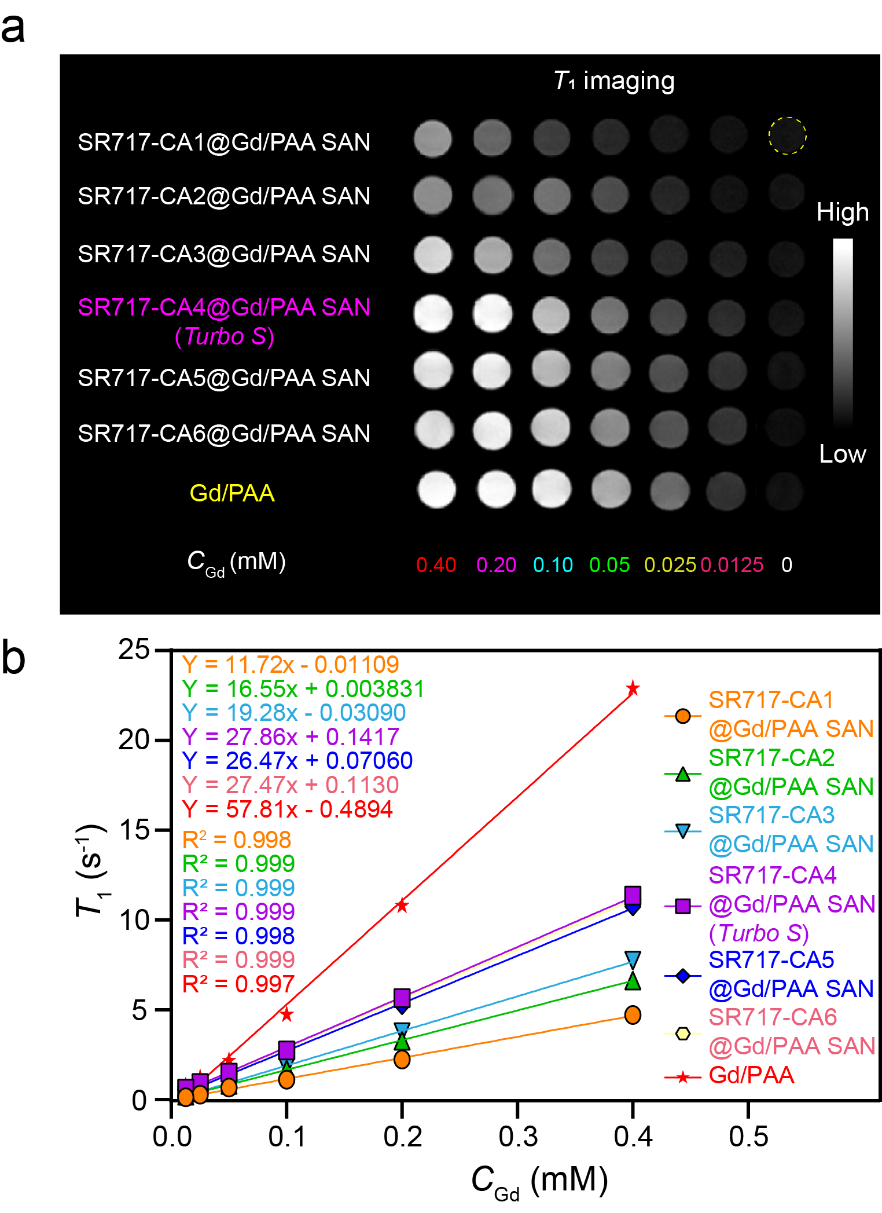


**Figure S1.** (a): *T*_1_-weighted MRI images of SR717-CA1-6@Gd/PAA SAN, and Gd/PAA at various Gd concentrations (0.40, 0.20, 0.10, 0.05, 0.025, 0.0125, or 0 mM) observed on a 3.0 T of clinical MRI scanner. (b): *T*_1_ relaxation rate (1/*T*_1_) plotted as a function of *C*_Gd_ for SR717-CA1-6@Gd/PAA SAN, and Gd/PAA.


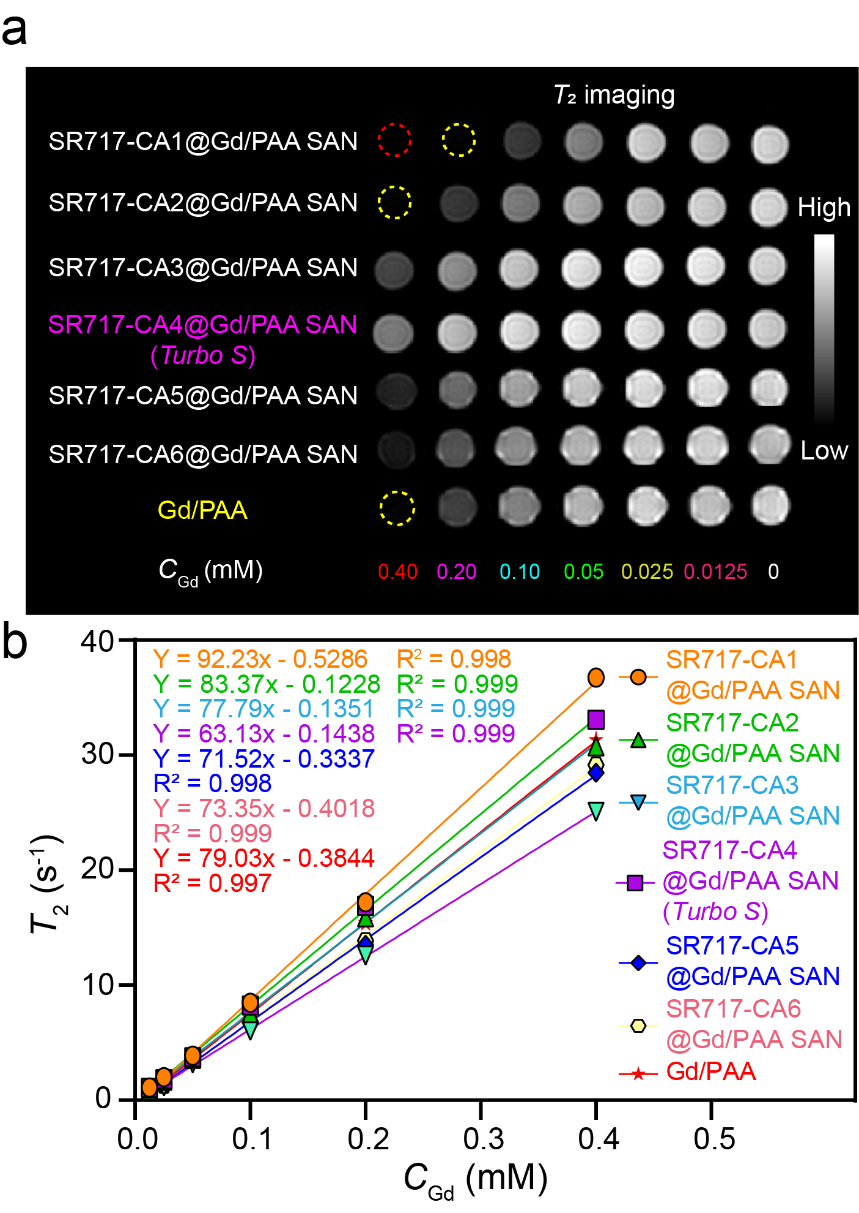


**Figure S2.** (a): *T*_2_-weighted MR images of SR717-CA1-6@Gd/PAA SAN, and Gd/PAA at various Gd concentrations (0.40, 0.20, 0.10, 0.05, 0.025, 0.0125, or 0 mM) observed on a 3.0 T of clinical MRI scanner. (b): *T*_2_ relaxation rate (1/*T*_2_) plotted as a function of *C*_Gd_ for SR717-CA1-6@Gd/PAA SAN, and Gd/PAA.

**
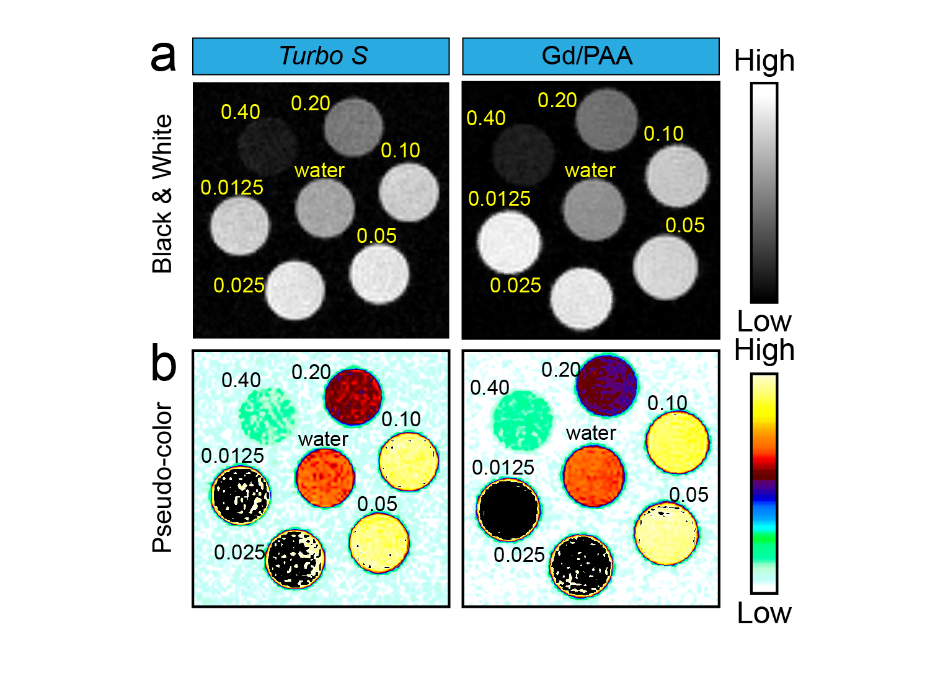
**

**Figure S3.** *T*_2_-weighted black & white (a) or pseudo-color (b) MR images of *Turbo S*, and Gd/PAA at various Gd concentrations (0.40, 0.20, 0.10, 0.05, 0.025, 0.0125, or 0 mM) observed on a 7.0 T of MRI scanner.

**
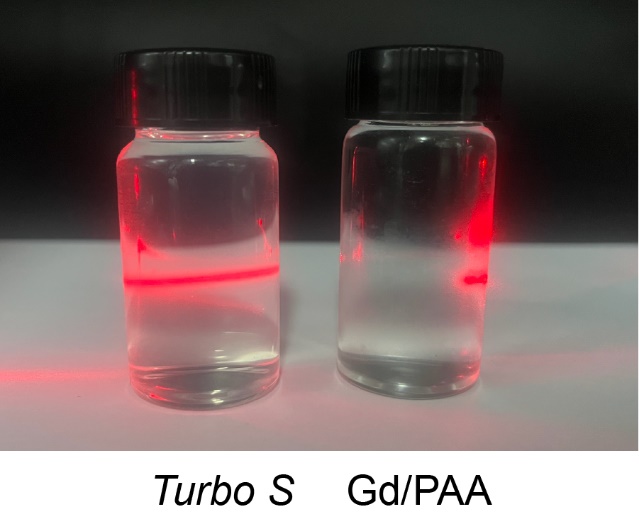
**

**Figure S4.** The situation of the Tyndall effect generated by *Turbo S*, and Gd/PAA under laser irradiation (650 nm).

**
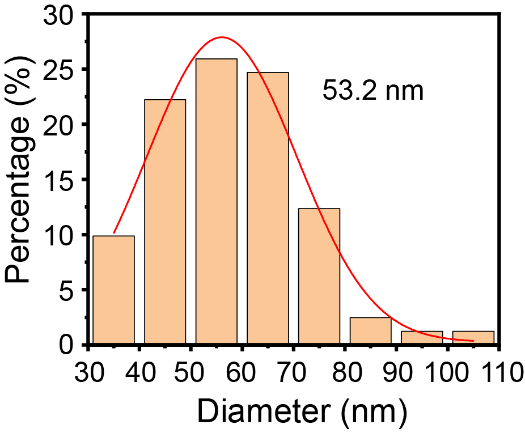
**

**Figure S5.** The size distribution of *Turbo S* obtained under TEM view.

**
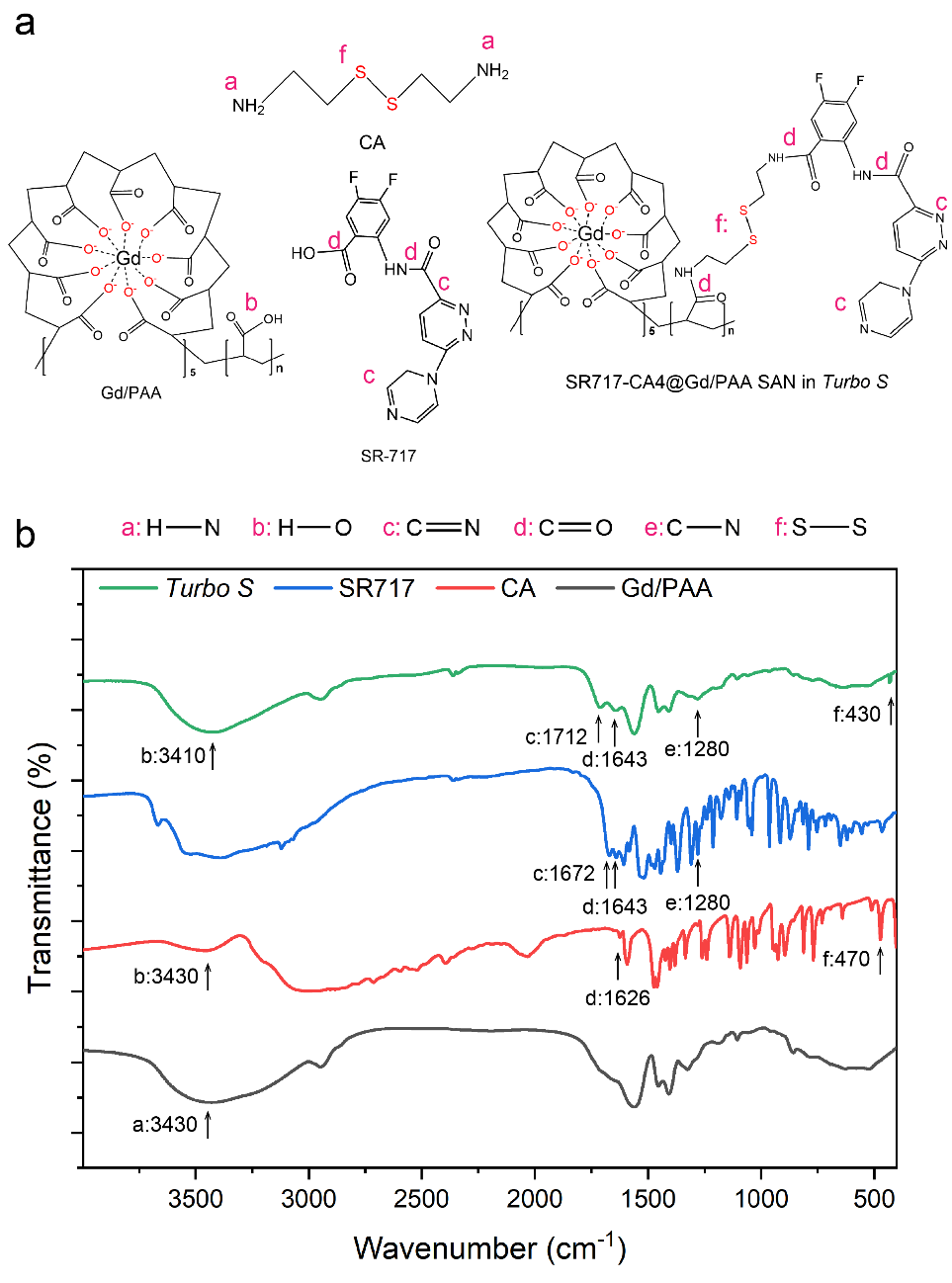
**

**Figure S6.** Molecular structures of the Gd/PAA, CA, SR717 and SR717-CA4@Gd/PAA SAN in *Turbo S* (a), and the corresponding Fourier transform infrared (FTIR) spectra (b).


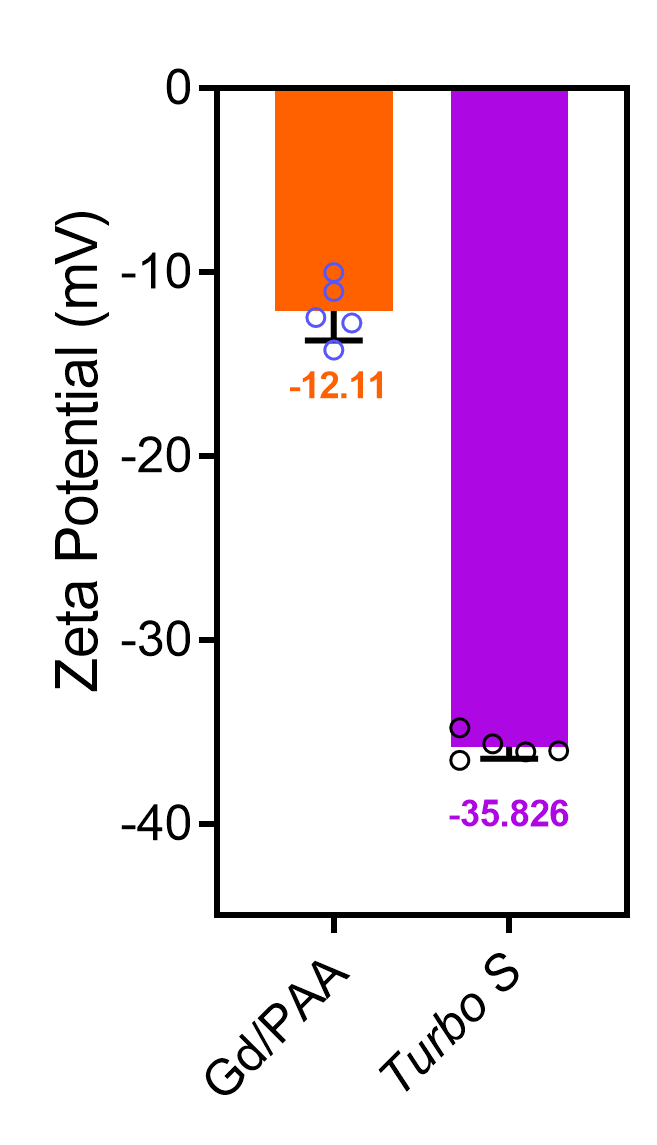


**Figure S7.** Zeta potential of Gd/PAA, and *Turbo S*. Mean ± S.D., *n* = 5.

**
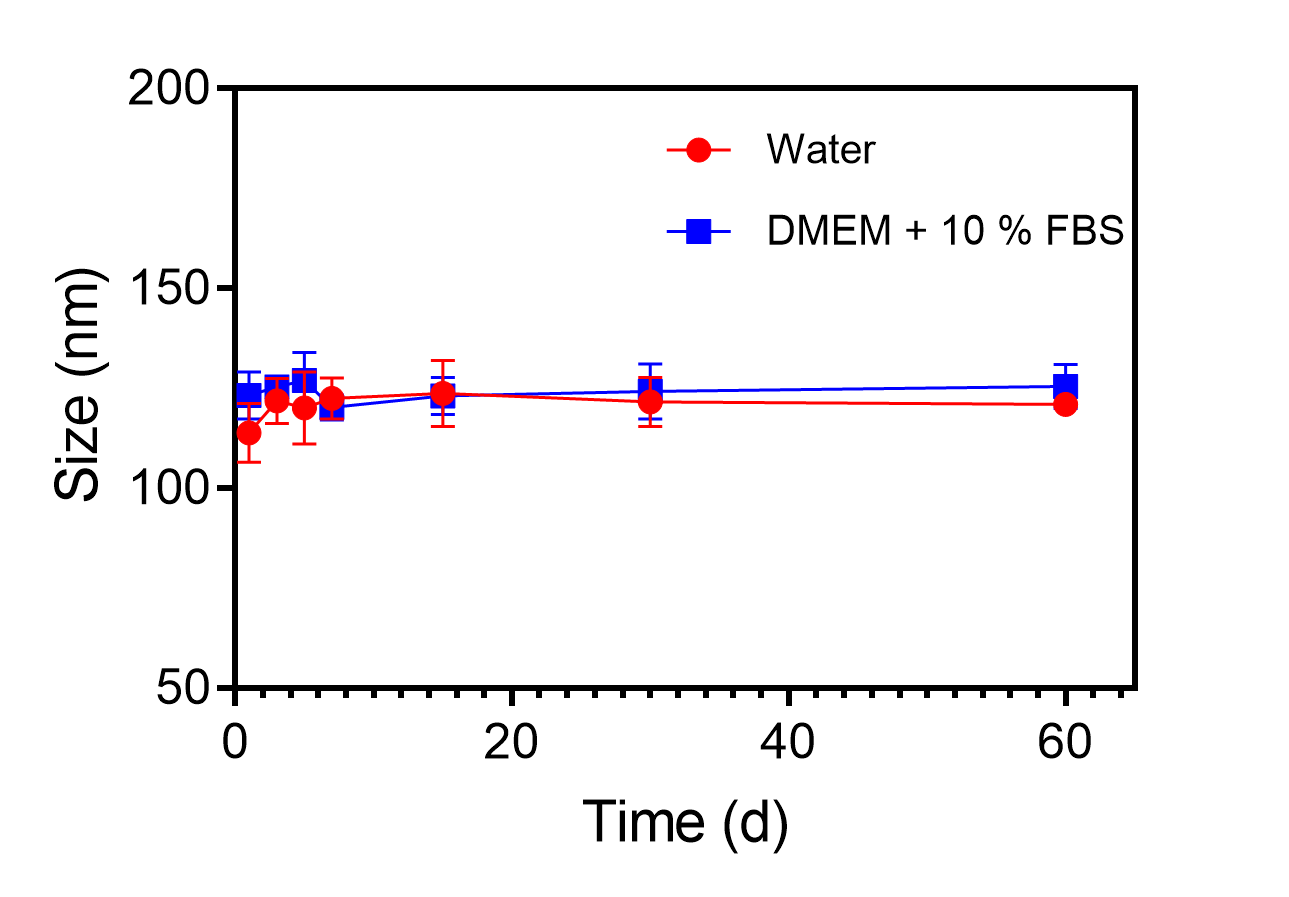
**

**Figure S8.** The hydrodynamic size of *Turbo S* in water or DMEM + 10 % FBS, measured by DLS after storage of different time. Mean ± S.D., *n* = 3.


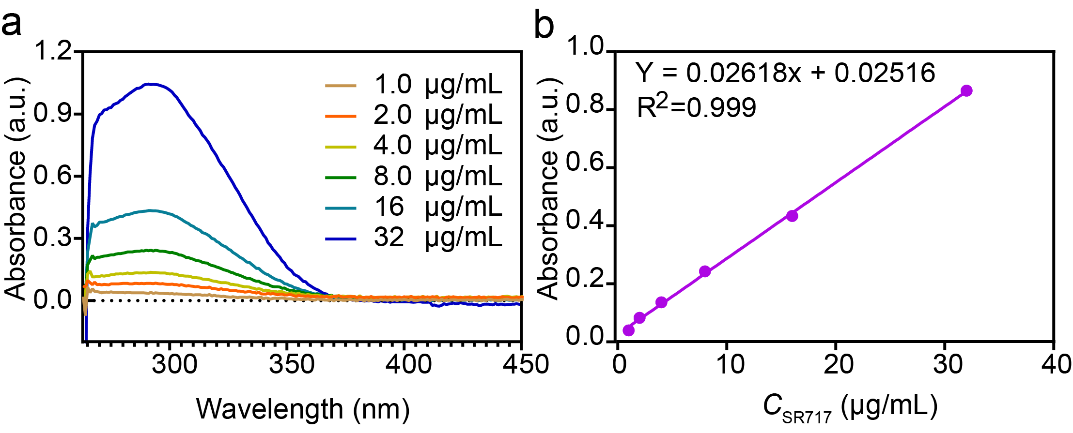


**Figure S9.** UV absorption spectra of SR717 solutions with different concentrations (1.0-32 μg/mL) in DMF (a), and the standard curve (b) fitted based on the absorbance at 294 nm for each concentration.


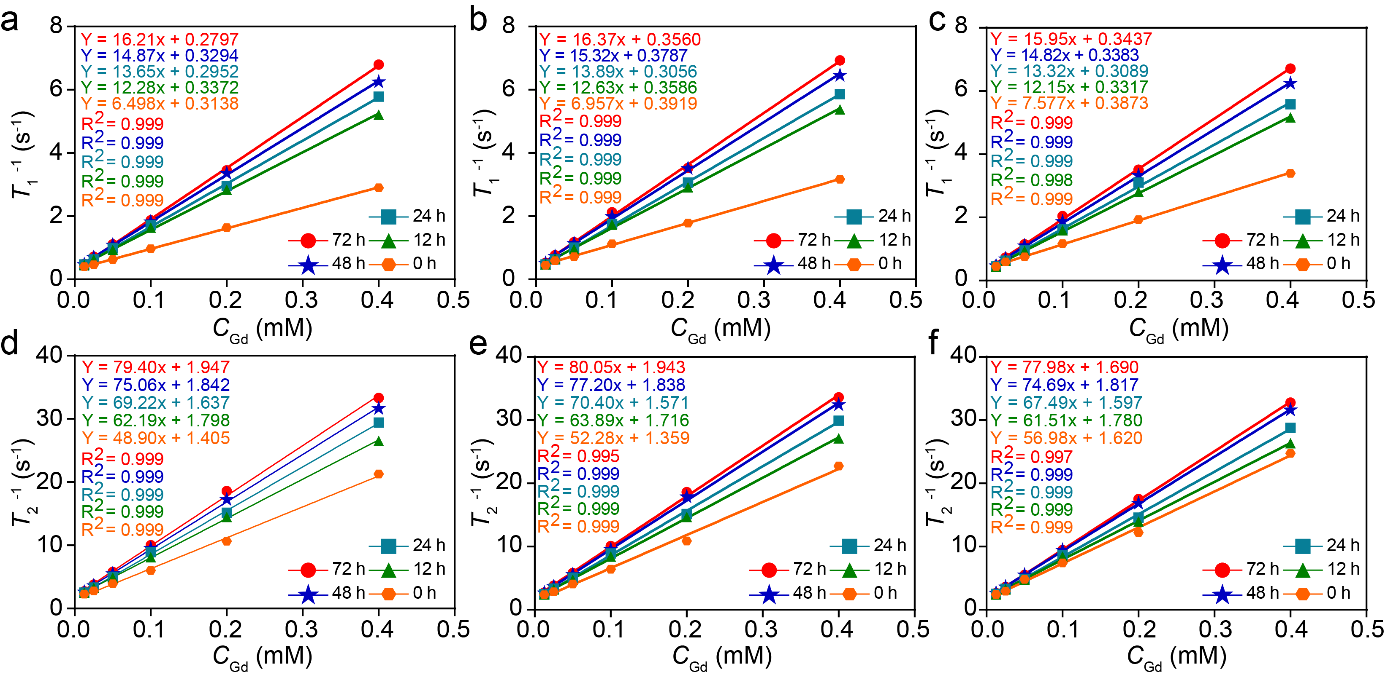


**Figure S10.** 1/*T*_1_ (a-c) and 1/*T*_2_ (d-f) of *Turbo S* measured on a 7.0 T MRI scanner after incubation in PBS with 10 mM of GSH for 0, 12, 24, 48, and 72 h in triplicate.


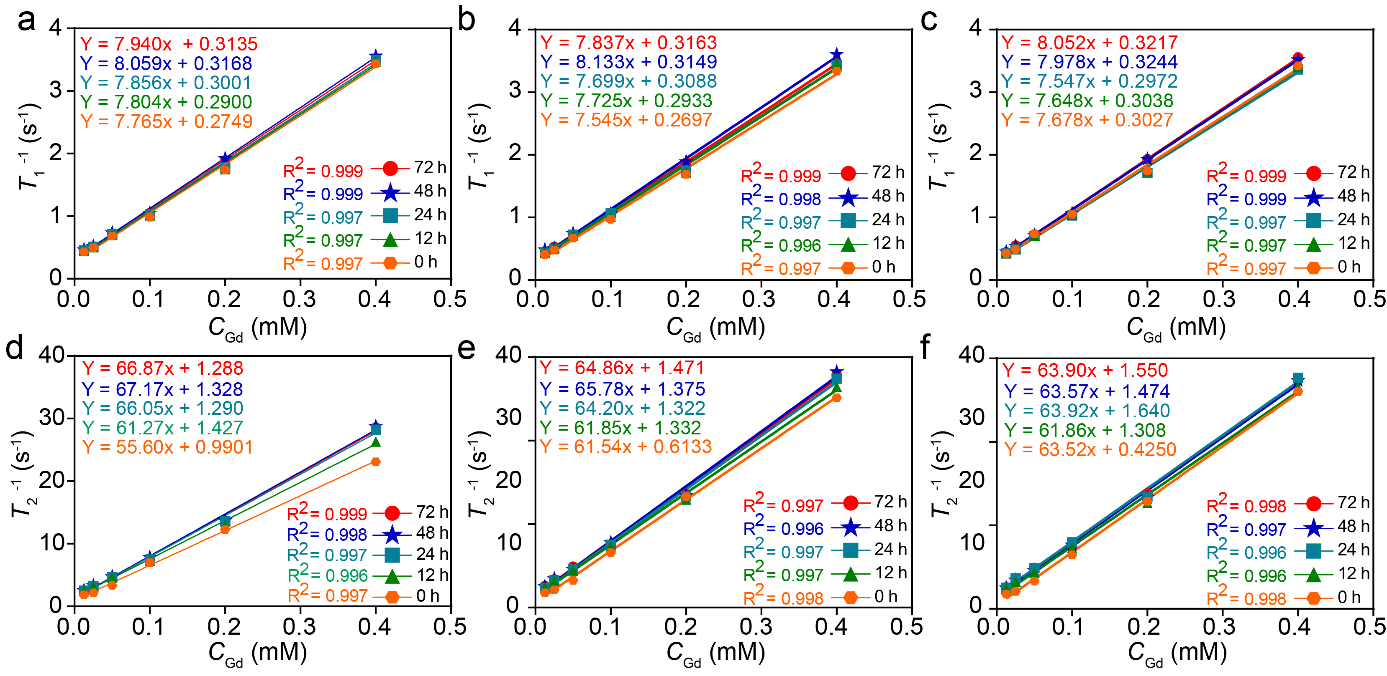


**Figure S11.** 1/*T*_1_ (a-c) and 1/*T*_2_ (d-f) of *Turbo S* measured on a 7.0 T MRI scanner after incubation in PBS without 10 mM of GSH for 0, 12, 24, 48, and 72 h in triplicate.


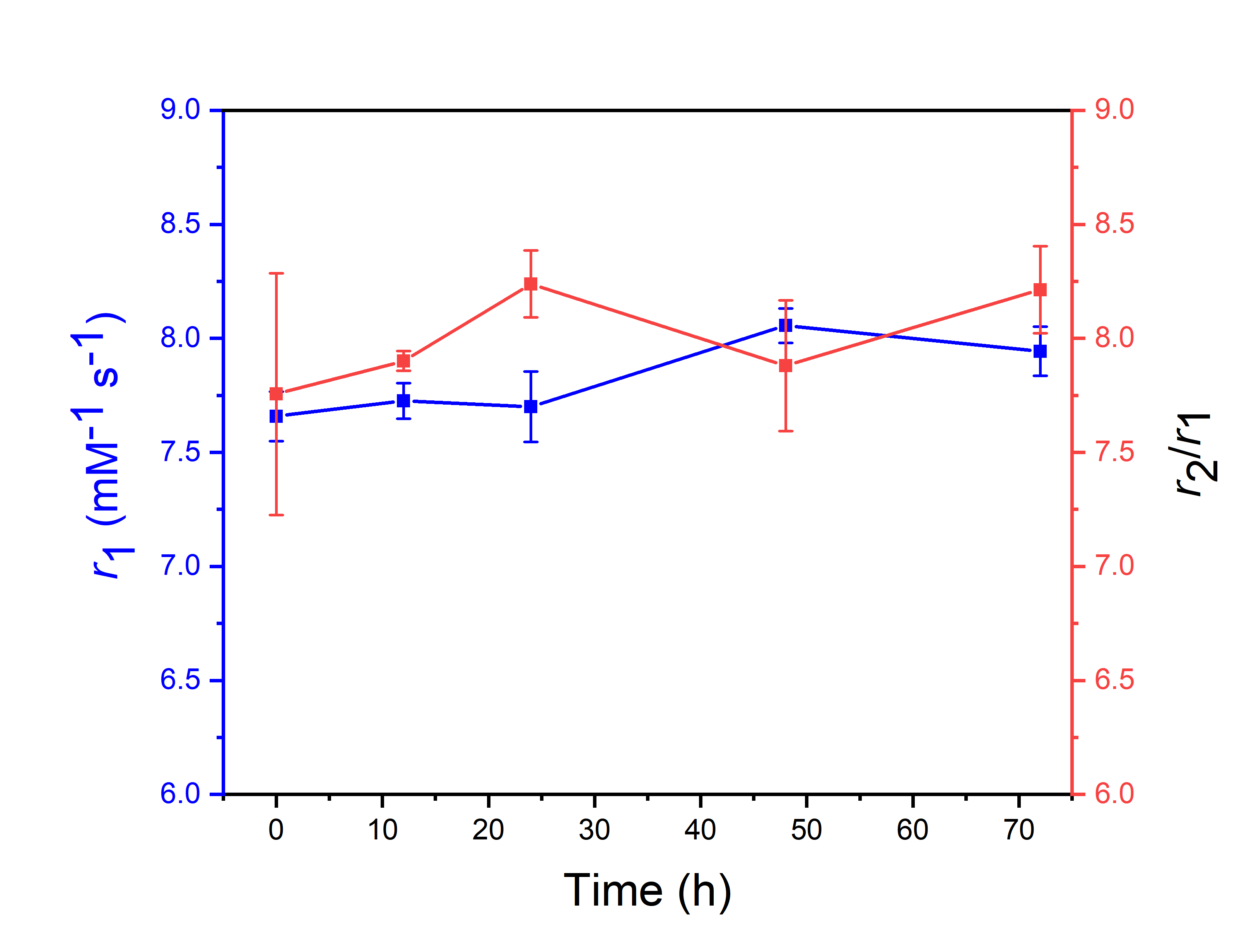


**Figure S12.** The changes of *r*_1_ value (blue curve) or *r*_2_/*r*_1_ ratio (red curve) of *Turbo S* measured on a 7.0 T MRI scanner after incubation in pH 7.4 of PBS without 10 mM of GSH at 37 ℃ for 0-72 h. Mean ± S.D., *n* = 3.


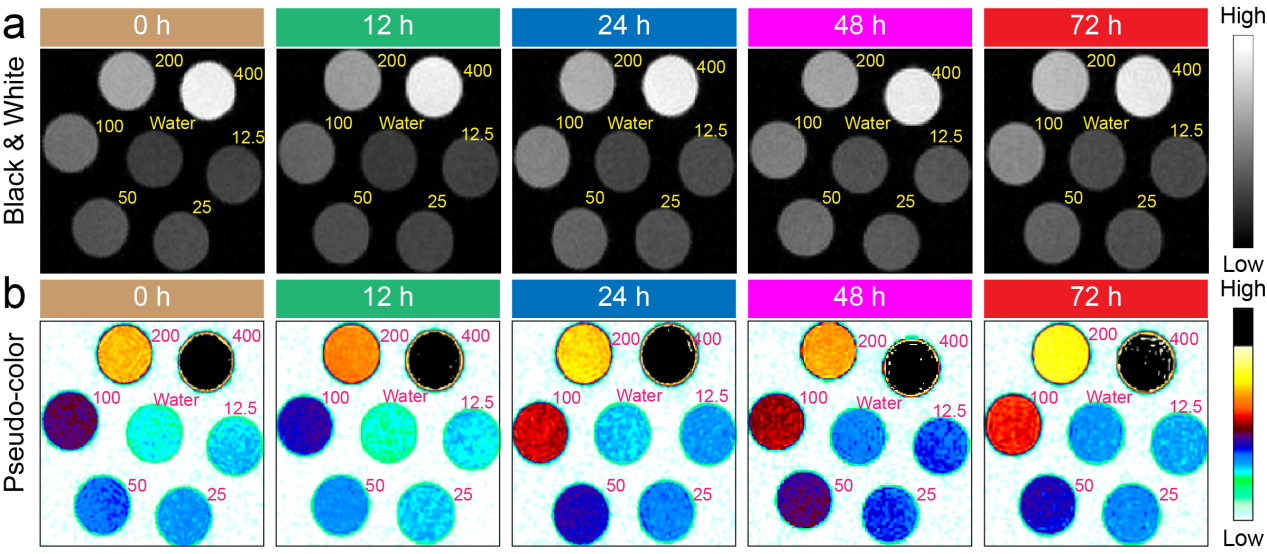


**Figure S13.** (a, b): The black & white (a), and the corresponding pseudo-colored (b) *T*_1_-weighted images of *Turbo S* with various *C*_Gd_ (mM) observed on a 7.0 T MRI scanner after incubation in PBS (pH 7.4) without 10 mM of GSH for 0, 12, 24, 48, and 72 h.


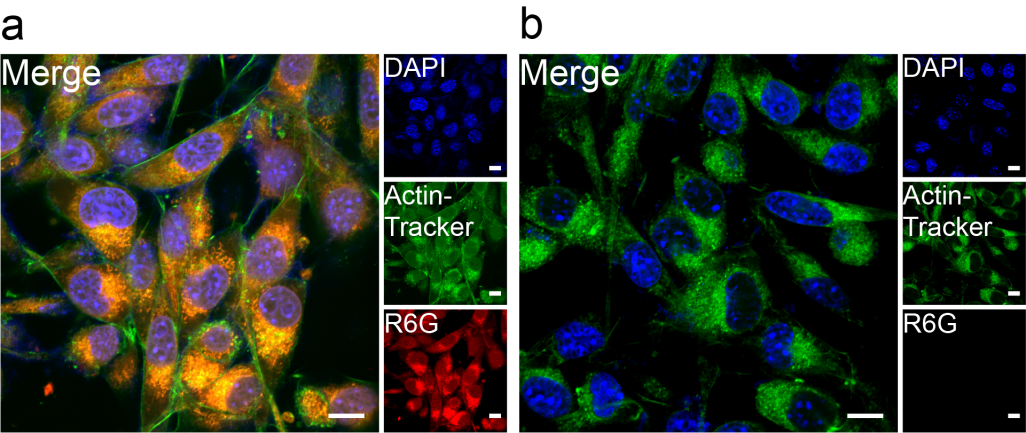


**Figure S14.** CLSM images of MC38 cells after incubation with R6G-*Turbo S* (red fluorescence) (a) or PBS (b) for 4.0 h, and staining with Actin-Tracker (green fluorescence for cytoskeleton) and DAPI (blue fluorescence for cell nuclei). Scale bar: 10 μm.


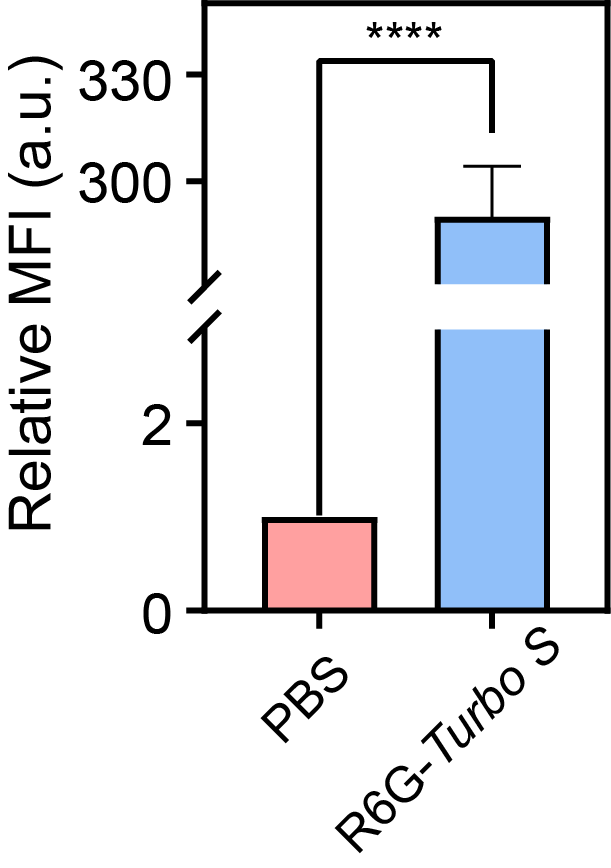


**Figure S15.** Fluorescence distributions quantitative analysis of 4T1 cells after treatment with R6G-*Turbo S* or PBS for 4.0 h measured by flow cytometry. **** P < 0.0001.


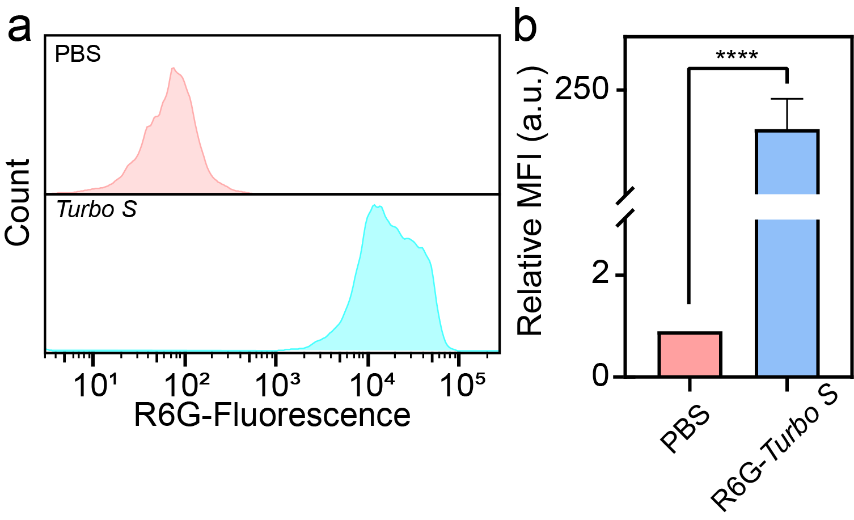


**Figure S16.** Fluorescence distributions (a) and the corresponding quantitative analysis (b) of MC38 cells after treatment with R6G-*Turbo S* or PBS for 4.0 h measured by flow cytometry. **** P < 0.0001.


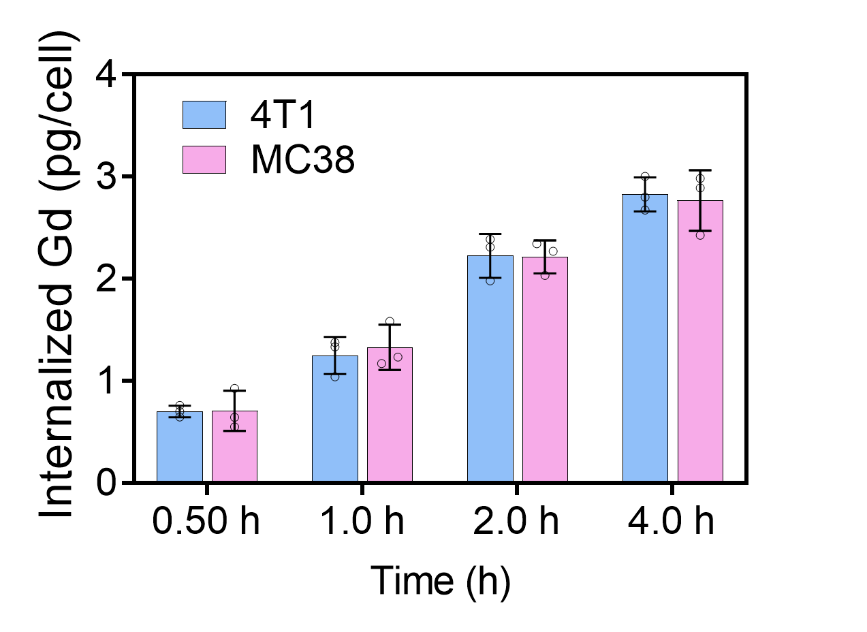


**Figure S17.** The internalized Gd dosage in 4T1, or MC38 cells after incubation with *Turbo S* for various time (0.50, 1.0, 2.0, or 4.0 h). Mean ± S.D., *n* = 3.


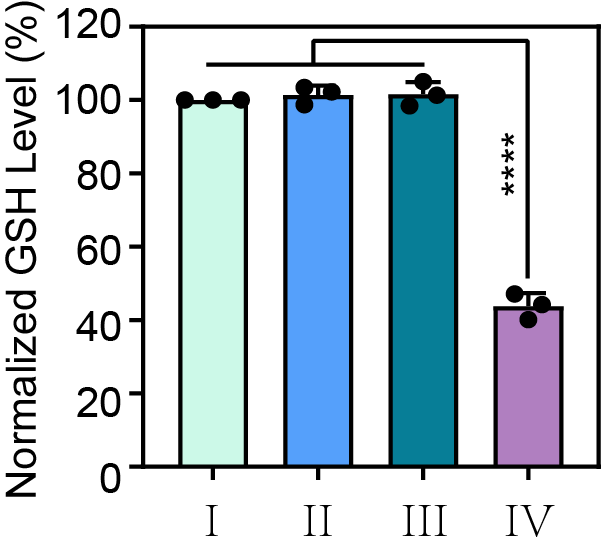


**Figure S18.** The relative intracellular GSH content in 4T1 cells after treatment with PBS (I), Gd/PAA (II), free SR717 (III), or *Turbo S* (IV) for 24 h. Mean ± S.D., *n* =3. **** P < 0.0001.


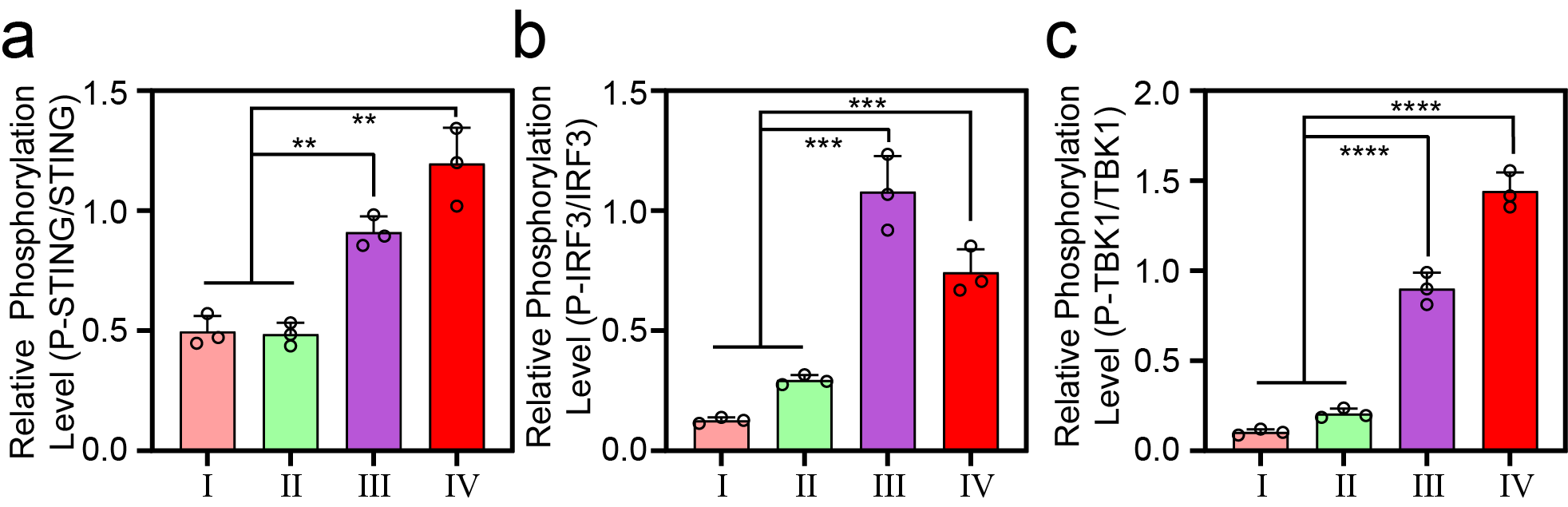


**Figure S19.** (a-c): The relative phosphorylation levels of P-STING/STING (a), P-IRF3/IRF3 (b), P-TBK1/TBK1 (c) in 4T1 cells after treatment with PBS (I), Gd/PAA (II), free SR717 (III), or *Turbo S* (IV) for 24 h, measured by western blot. Mean ± S.D., *n* =3. ** P < 0.01, *** P < 0.001, **** P < 0.0001.


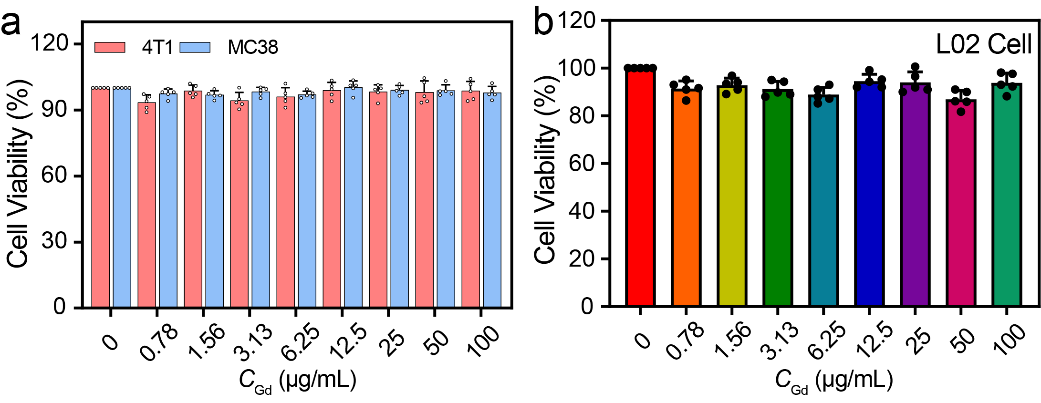


**Figure S20.** (a, b): Cell viabilities of 4T1, MC38 (a), or L02 cells (b) after treatment with Gd/PAA for 24 h, measured by the MTT assay. Mean ± S.D., *n* = 5.


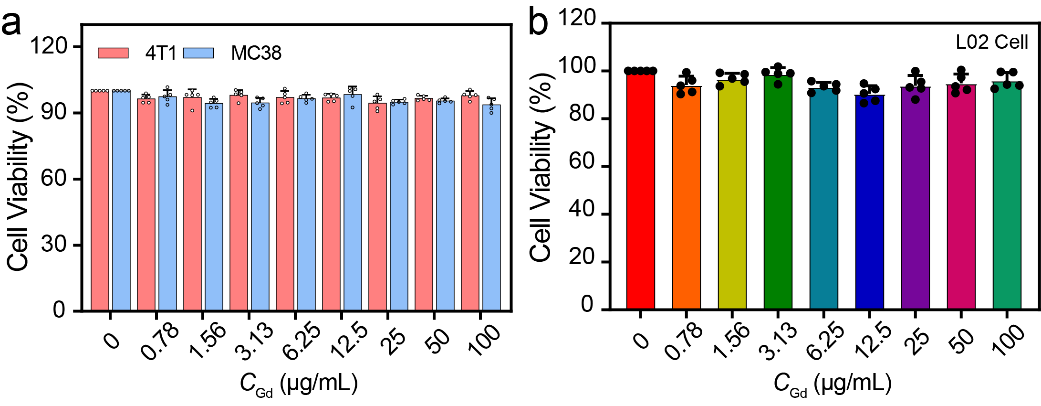


**Figure S21.** (a, b): Cell viabilities of 4T1, MC38 (a), or L02 cells (b) after treatment with *Turbo S* for 24 h, measured by the MTT assay. Mean ± S.D., *n* = 5.

**
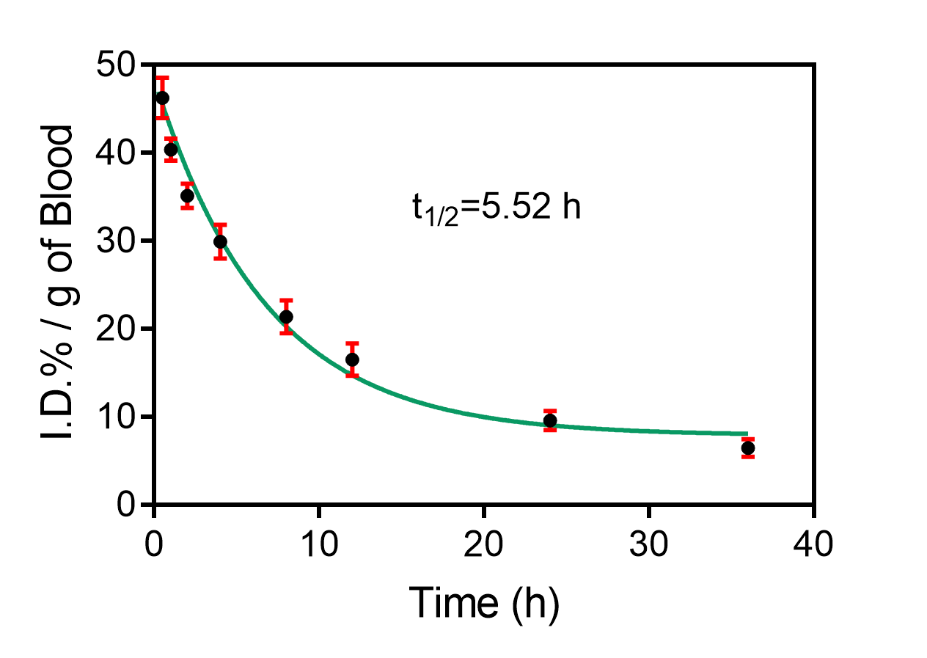
**

**Figure S22.** The pharmacokinetics of Gd level in the blood of 4T1 tumor-bearing Balb/c mice after intravenous injection of *Turbo S* (*C*_Gd_ = 5.0 mg/kg). Mean ± S.D., *n* = 3.


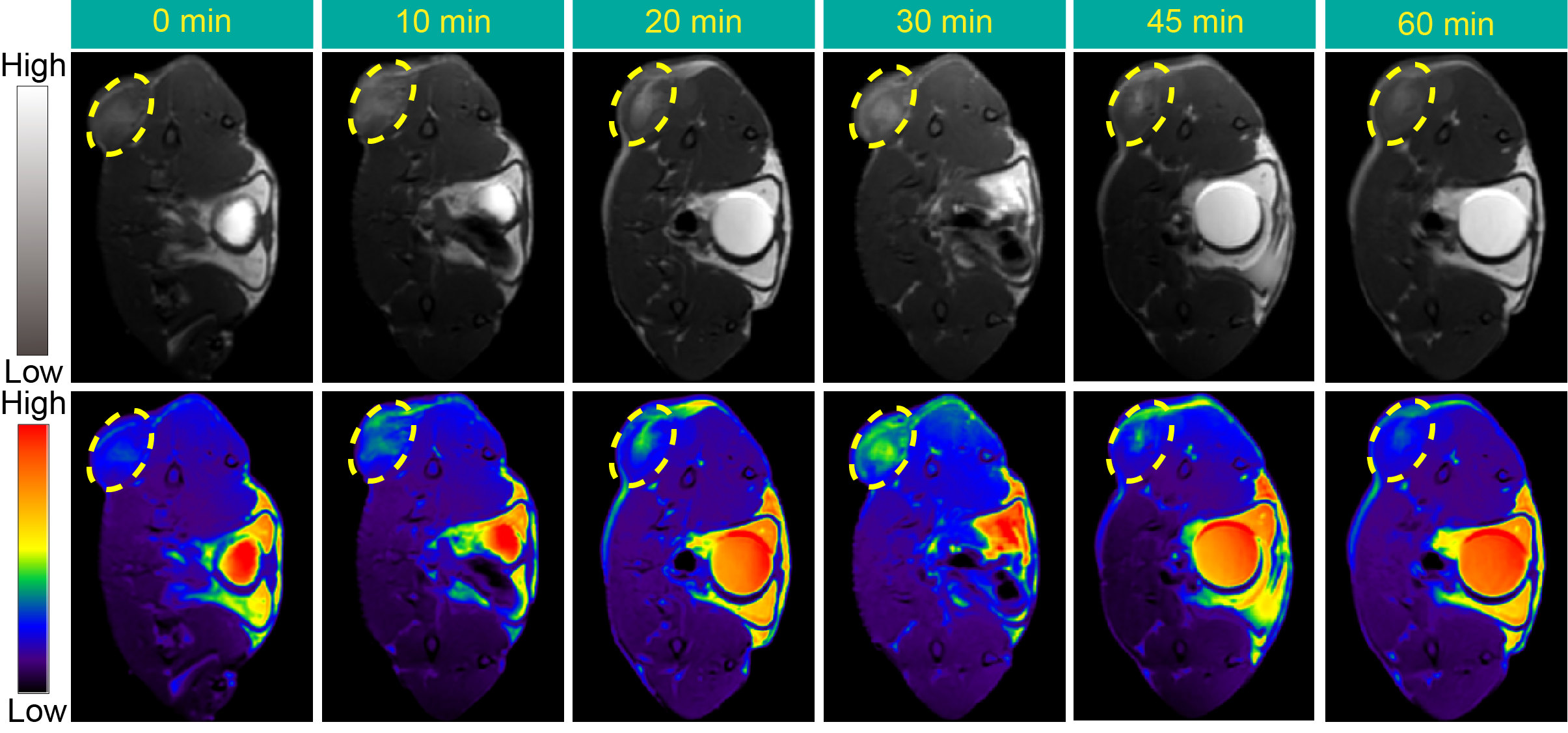


**Figure S23.** Black & white, and the corresponding pseudo-color images of 4T1 tumor-bearing Balb/c mice pre-injection (*i.e.*, 0 min), or at 10, 20, 30, 45, and 60 min post-injection (*i.v.*) of Gadovist^®^ (*C*_Gd_ = 5.0 mg/kg) observed on a clinical 3.0 T MRI scanner.


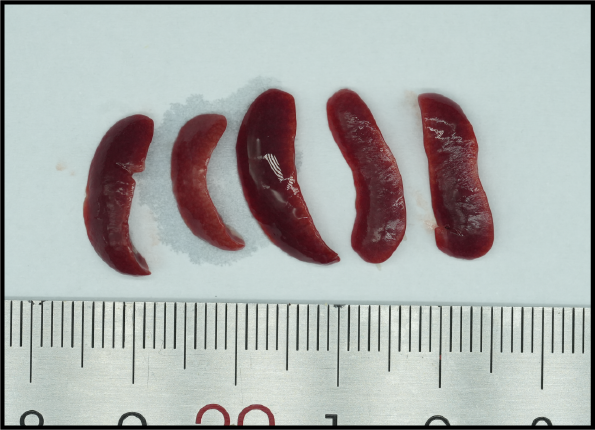


**Figure S24.** Photos of the spleen of healthy mice, *n* = 5.


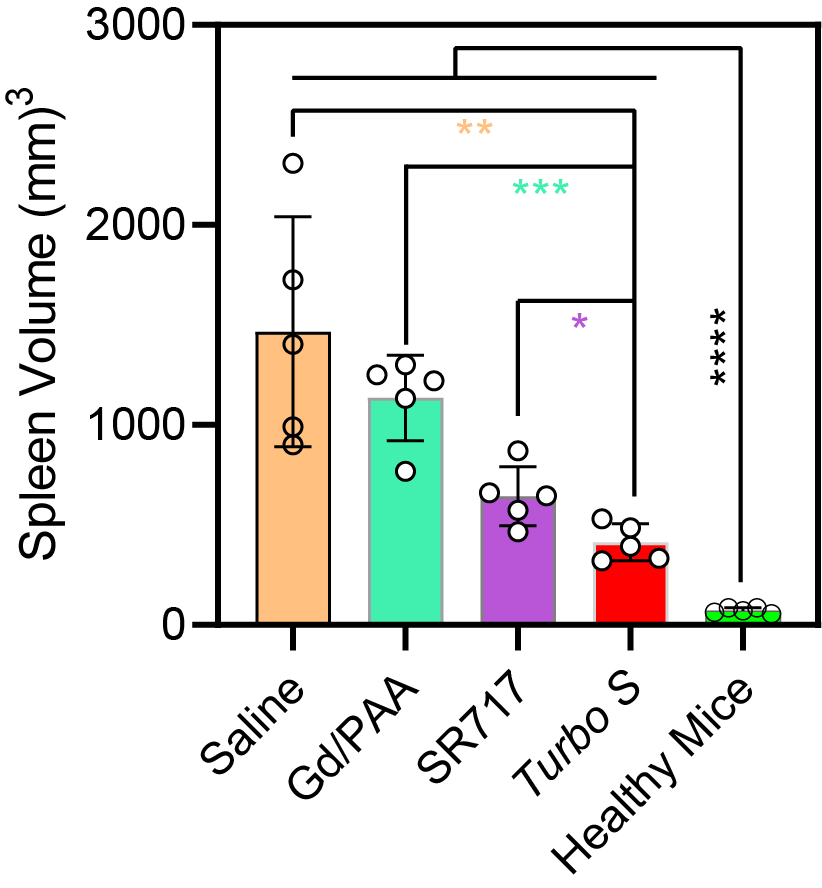


**Figure S25.** Spleen volumes of healthy mice and 4T1 tumor-bearing mice after treatment with Saline, Gd/PAA (Gd dosage: 5.0 mg/kg.), free SR717 (SR717 dosage: 30 mg/kg), or *Turbo S* (Gd dosage: 5.0 mg/kg, SR717 dosage: 8.9 mg/kg) for 14 days (*i.v.* injection)*.* Mean ± S.D., *n* = 5. * P < 0.05, ** P < 0.01, *** P < 0.001, **** P < 0.0001.


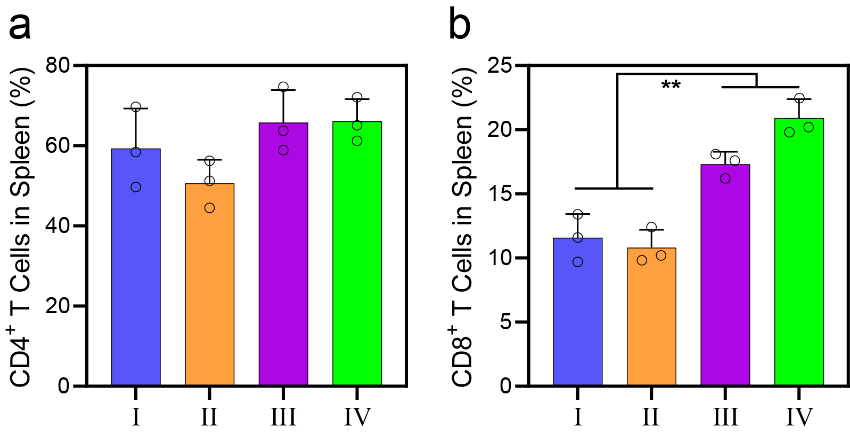


**Figure S26.** (a, b): Expression of CD4^+^ T cells (a) and CD8^+^ T cells (b) in spleen of mice after *i.v.* injection of saline (I), Gd/PAA (II, Gd dosage: 5.0 mg/kg), free SR717 (III, SR717 dosage: 30 mg/kg), or *Turbo S* (IV, Gd dosage: 5.0 mg/kg, SR717 dosage: 8.9 mg/kg). Mean ± S.D., *n* = 3. ** P < 0.01.


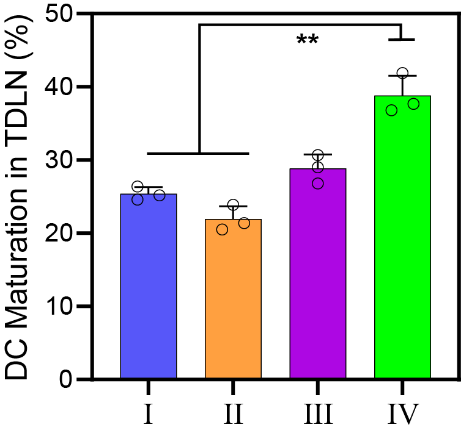


**Figure S27.** Expression of DCs in the tumor-draining lymph nodes (TDLN) of mice after *i.v.* injection of saline (I), Gd/PAA (II, Gd dosage: 5.0 mg/kg), free SR717 (III, SR717 dosage: 30 mg/kg), or *Turbo S* (IV, Gd dosage: 5.0 mg/kg, SR717 dosage: 8.9 mg/kg). Mean ± S.D., *n* = 3. ** P < 0.01.


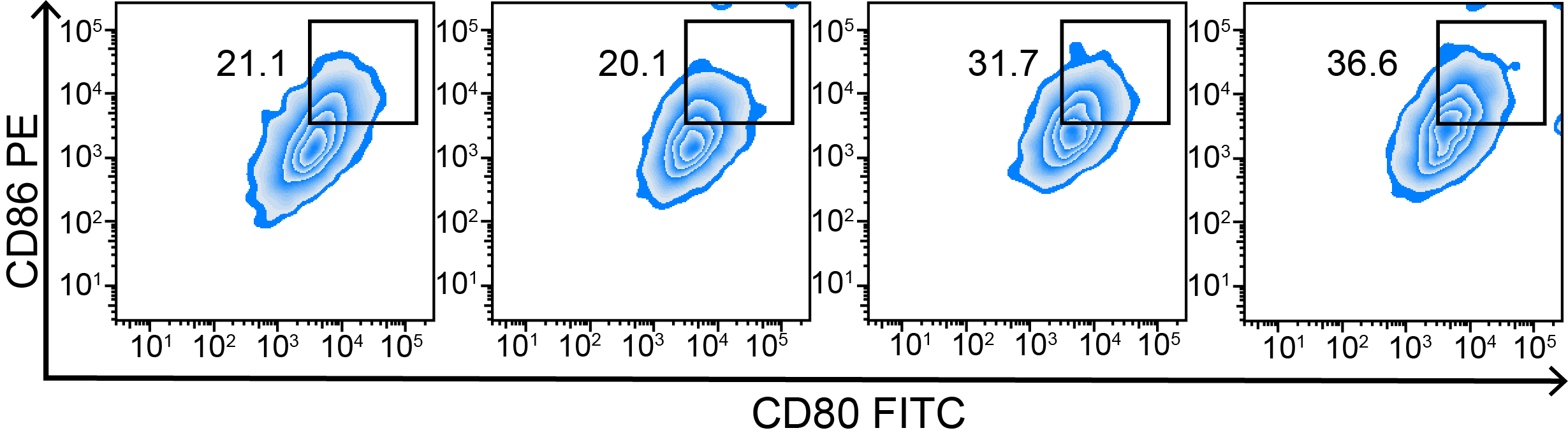


**Figure S28.** Expression of DCs (CD11c^+^-APC, CD80^+^-FITC, CD86^+^-PE) in the tumor of mice after *i.v.* injection of saline (I), Gd/PAA (II, Gd dosage: 5.0 mg/kg), free SR717 (III, SR717 dosage: 30 mg/kg), or *Turbo S* (IV, Gd dosage: 5.0 mg/kg, SR717 dosage: 8.9 mg/kg).


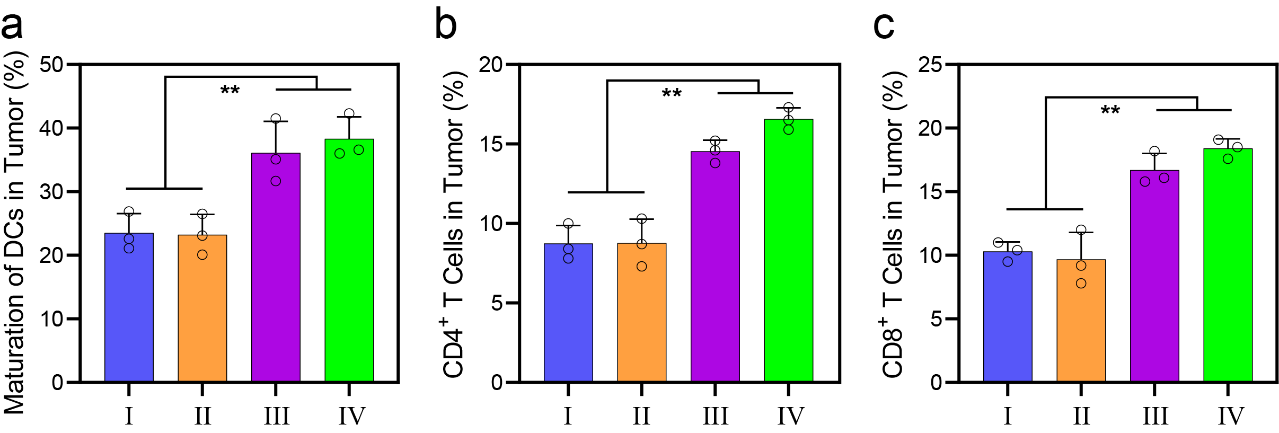


**Figure S29.** (a-c): Expression of DCs (a), CD4^+^T cells (b), and CD8^+^ T cells in the tumor of mice after *i.v.* injection of saline (I), Gd/PAA (II, Gd dosage: 5.0 mg/kg), free SR717 (III, SR717 dosage: 30 mg/kg), or *Turbo S* (IV, Gd dosage: 5.0 mg/kg, SR717 dosage: 8.9 mg/kg). Mean ± S.D., *n* = 3. ** P < 0.01.


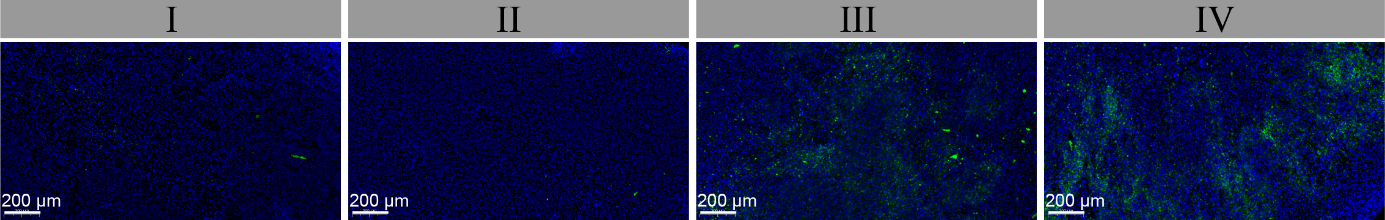


**Figure S30.** Representative optical microscope images of tumor tissue sections stained immunofluorescence after treatments of the group I (saline), II (Gd/PAA), III (free SR717), or IV (*Turbo S*). Blue: DAPI for cell nuclei; green: FITC for CD49b^+^ cells (NK cells).


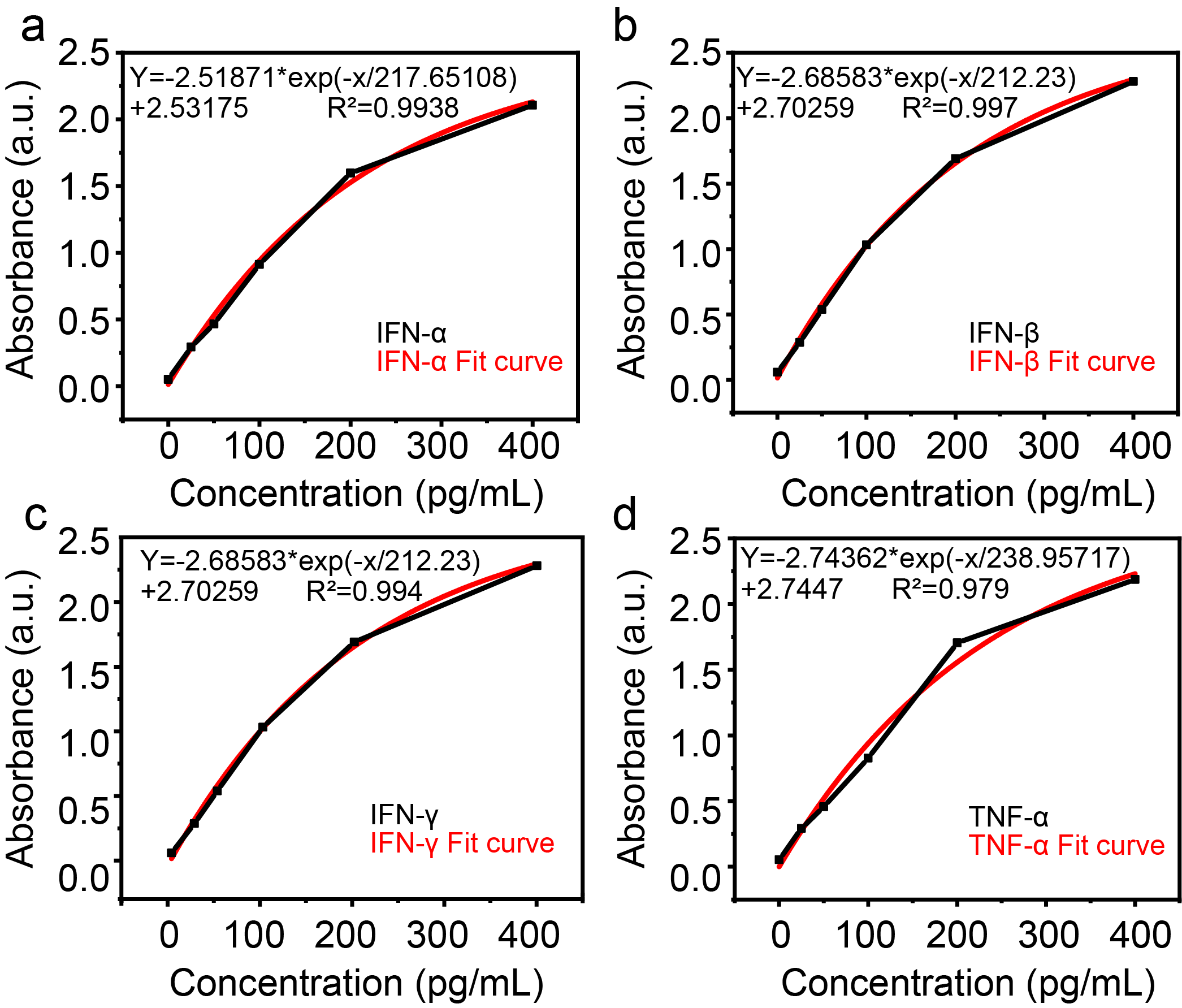


**Figure S31.** Standard curves of INF-α (a), INF-β (b), INF-γ (c) and TNF-α (d) obtained from the corresponding test Kit.


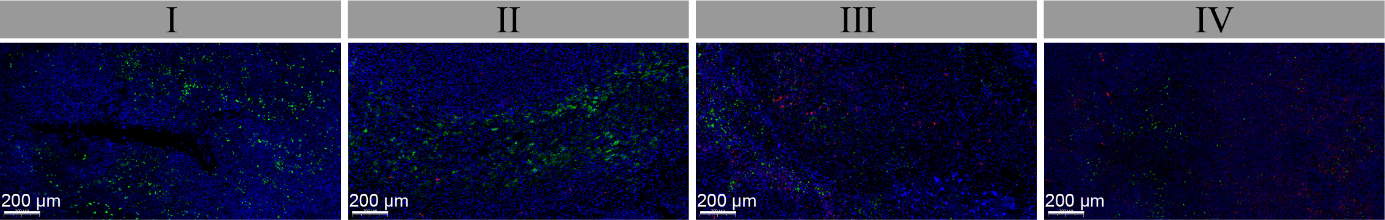


**Figure S32.** Representative optical microscope images of tumor tissue sections stained immunofluorescence after treatments of the group I (saline), II (Gd/PAA), III (free SR717), or IV (*Turbo S*). Blue: DAPI for cell nuclei; green: FITC for CD206^+^ cells (M2 macrophage), red: Cy3 for CD86^+^ cells (M1 macrophage).


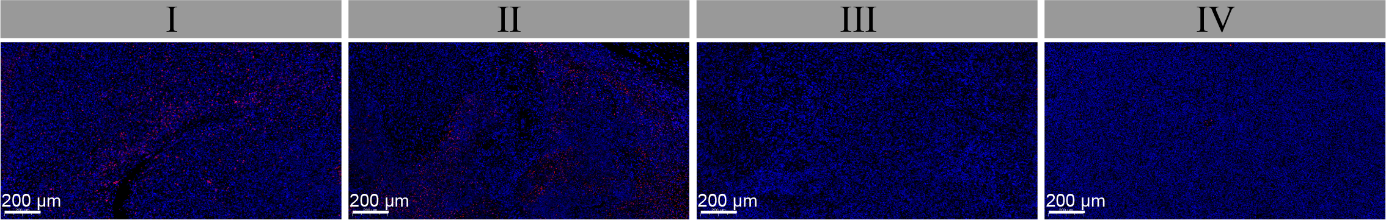


**Figure S33.** Representative optical microscope images of tumor tissue sections stained immunofluorescence after treatments of the group I (saline), II (Gd/PAA), III (free SR717), or IV (*Turbo S*). Blue: DAPI for cell nuclei; red: Cy3 for GR-1^+^ cells (MDSC).


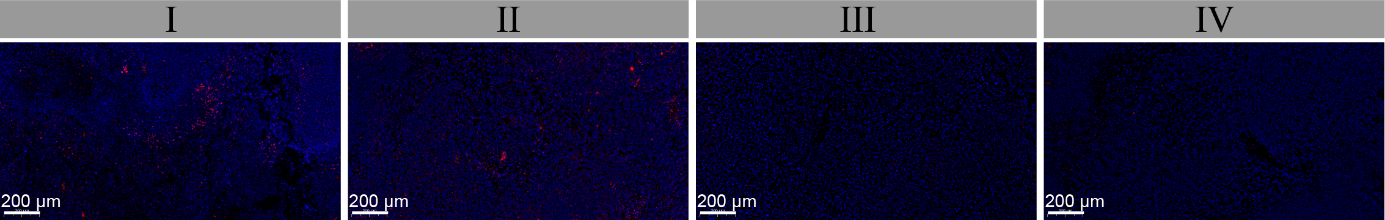


**Figure S34.** Representative optical microscope images of tumor tissue sections stained immunofluorescence after treatments of the group I (saline), II (Gd/PAA), III (free SR717), or IV (*Turbo S*). Blue: DAPI for cell nuclei; red: Cy3 for FoxP3^+^ cells (Treg).


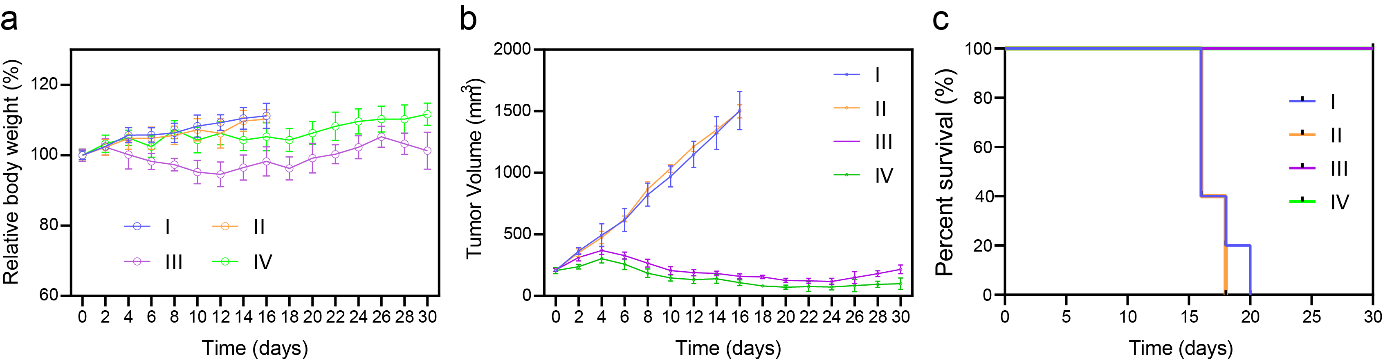


**Figure S35.** (a-c): Relative body weight (a), tumor volumes (b), or survival rates (c) of CT26 tumor-bearing mice after *i.v.* injection of saline (I), Gd/PAA (II, Gd dosage: 5.0 mg/kg), free SR717 (III, SR717 dosage: 30 mg/kg), or *Turbo S* (IV, Gd dosage: 5.0 mg/kg, SR717 dosage: 8.9 mg/kg). Mean ± S.D., *n* = 5.


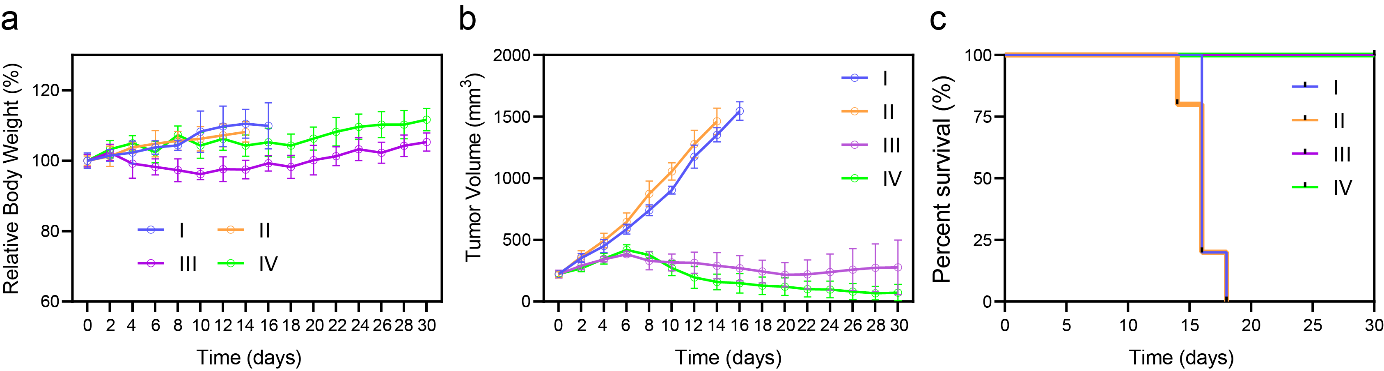


**Figure S36.** (a-c): Relative body weight (a), tumor volumes (b), or survival rates (c) of MC38 tumor-bearing mice after *i.v.* injection of saline (I), Gd/PAA (II, Gd dosage: 5.0 mg/kg), free SR717 (III, SR717 dosage: 30 mg/kg), or *Turbo S* (IV, Gd dosage: 5.0 mg/kg, SR717 dosage: 8.9 mg/kg). Mean ± S.D., *n* = 5.


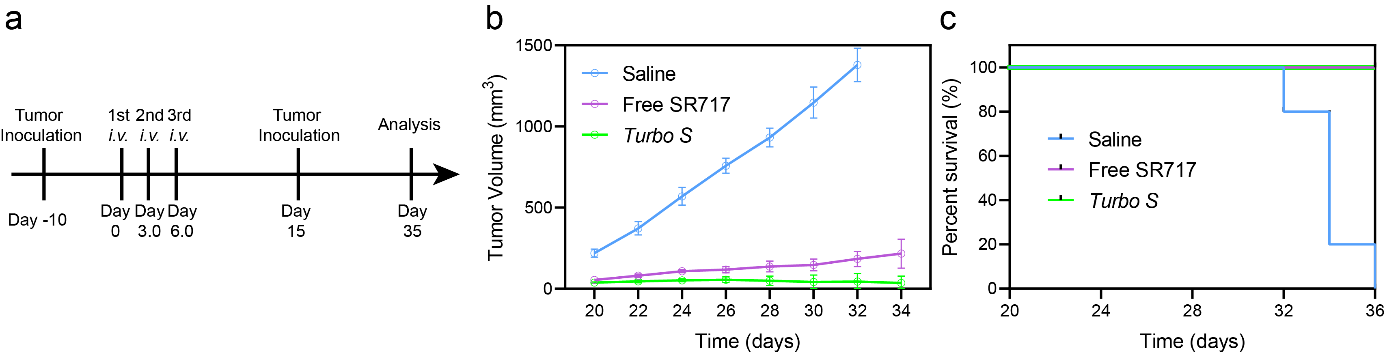


**Figure S37.** (a): Schematic illustration of the re-challenge studies. (b, c): Tumor volumes (b), or survival rates (c) of 4T1 re-challenged tumor-bearing mice.


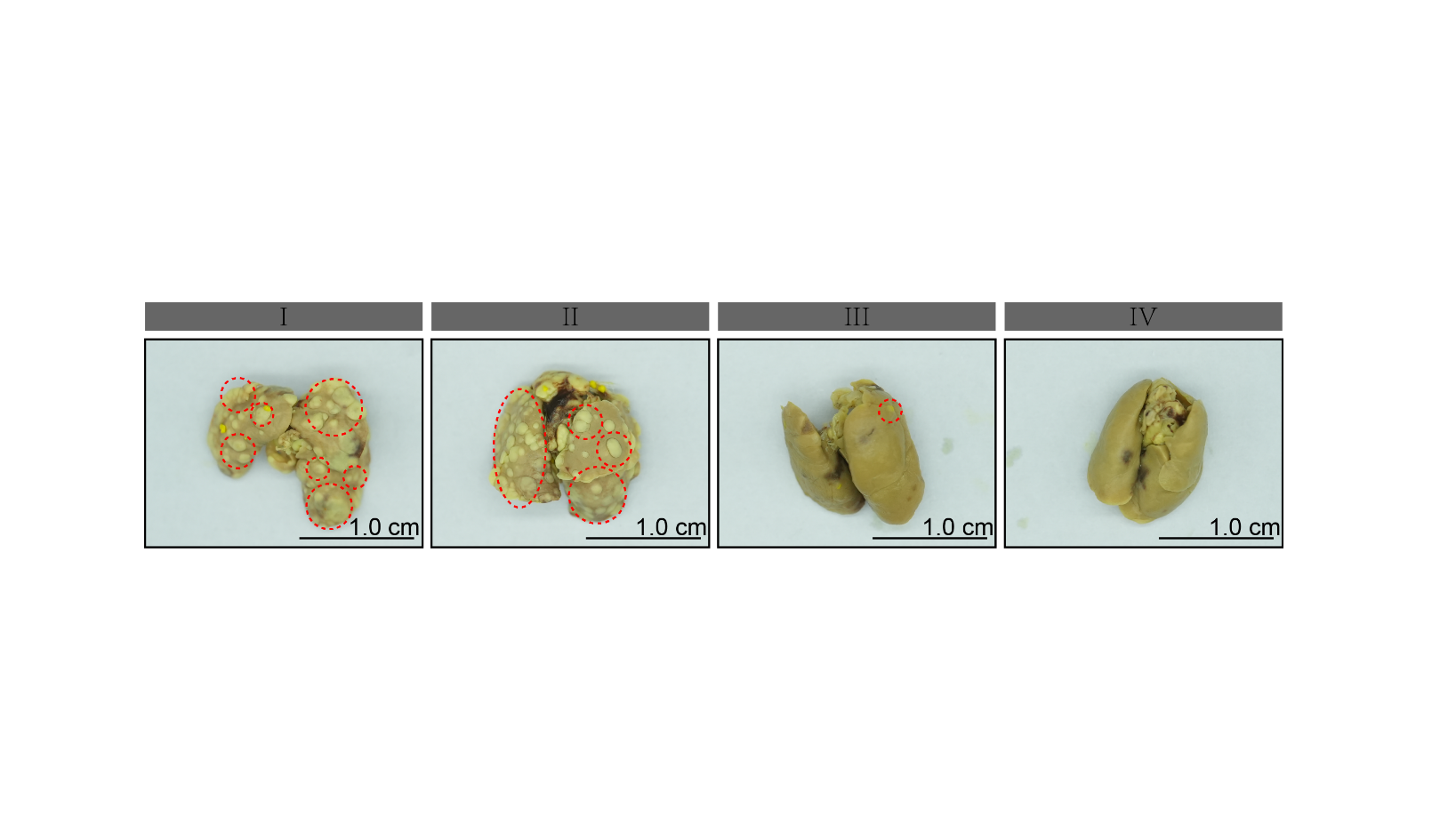


**Figure S38.** The lung metastasis status of tumors after treatment (*i.v.* injection) with saline (I), Gd/PAA (II, Gd dosage: 5.0 mg/kg.), free SR717 (III, SR717 dosage: 30 mg/kg), or *Turbo S* (IV, Gd dosage: 5.0 mg/kg, SR717 dosage = 8.9 mg/kg) for 12 days. All the lungs are stained with Bouin’s fluid, and the obvious tumor metastasis lesions are shown in the red circle.


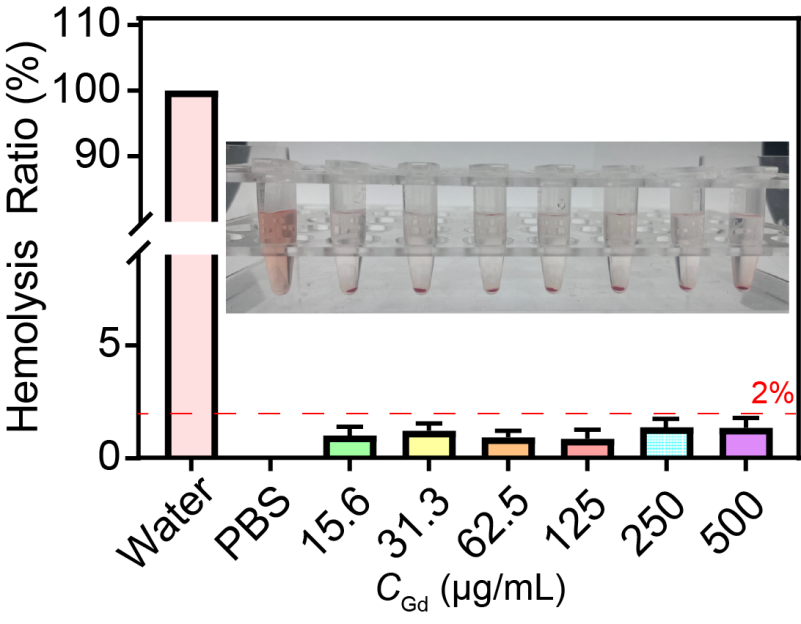


**Figure S39.** Quantitative analysis of hemolysis rates of ultra-pure water, PBS, and *Turbo S* under different *C*_Gd_ (15.60, 31.30, 62.50, 125, 250, or 500 μg/mL) conditions, accompanied by corresponding images of hemolysis. Mean ± S.D., *n* = 3.


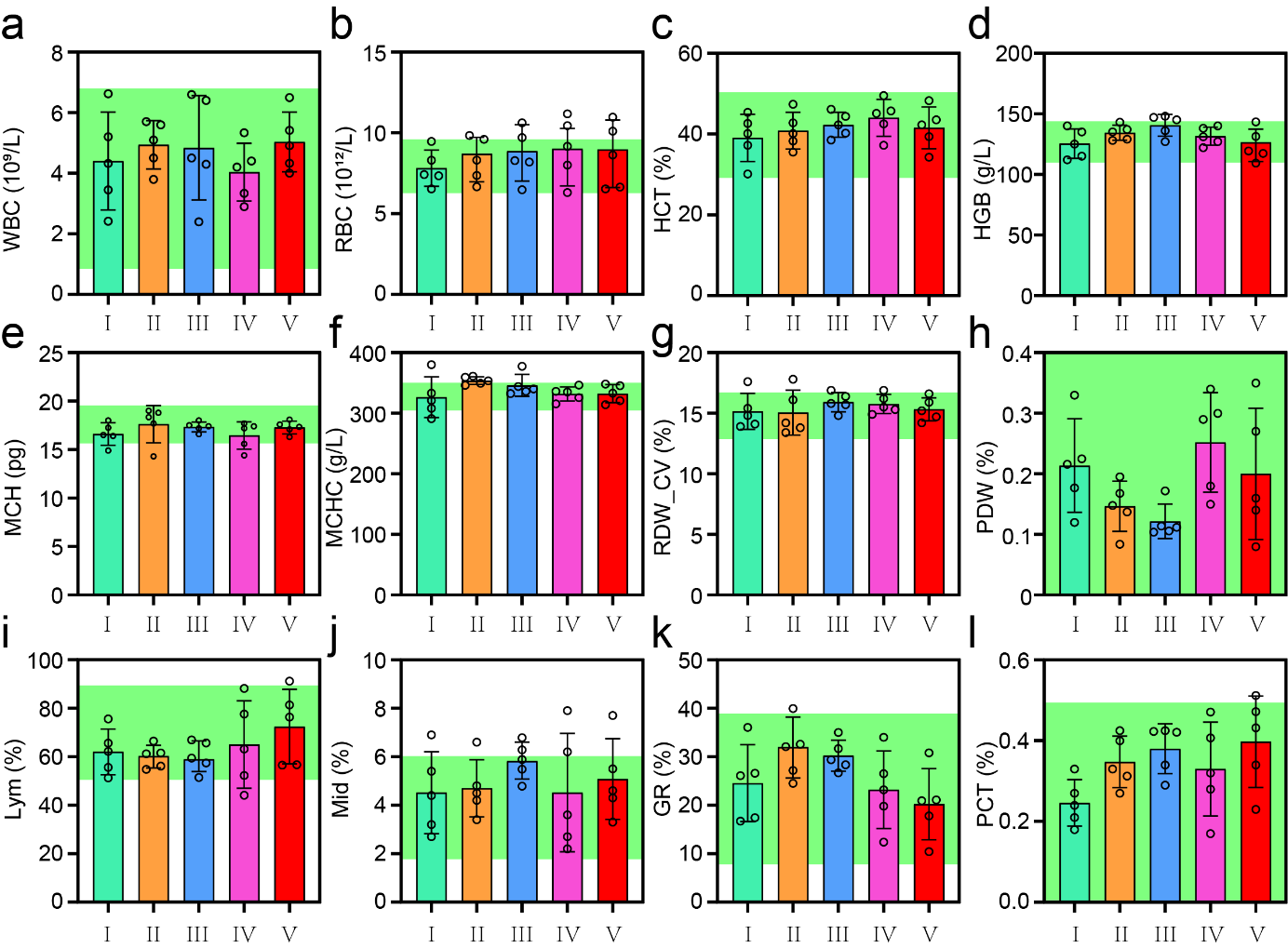


**Figure S40.** Hematology analysis of healthy mice without any treatment (I), or that after treatment with saline (II), Gd/PAA (Gd dosage: 5.0 mg/kg) (III), free SR717 (SR717 dosage: 30 mg/kg) (IV), or *Turbo S* (Gd dosage = 5.0 mg/kg, SR717 dosage = 8.9 mg/kg) (V) (a-l). Mean ± S.D., *n* = 5.


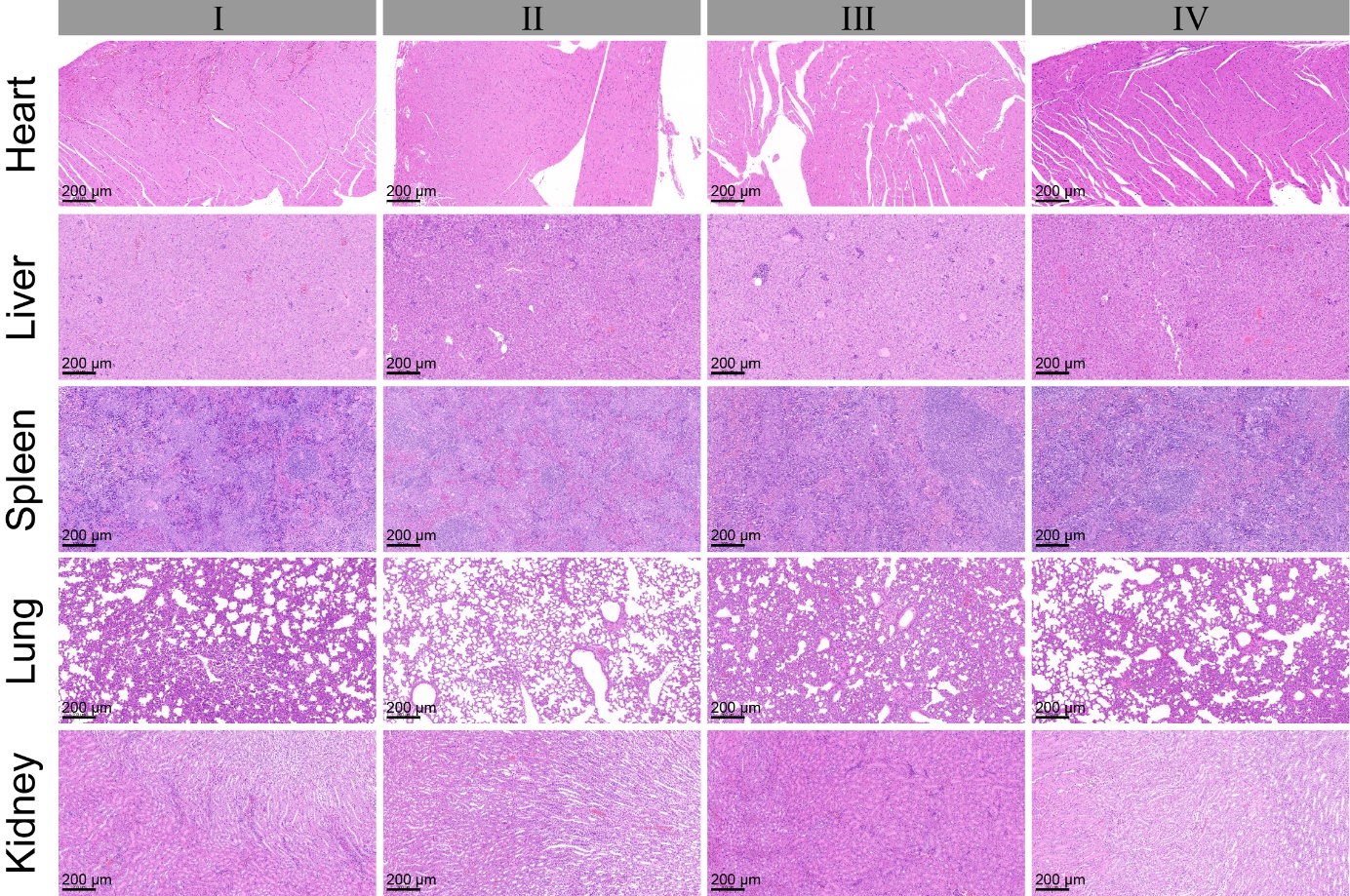


**Figure S41.** Representative optical microscopic images of H&E-stained major organs (heart, liver, spleen, lung, and kidney) for the tumor-bearing mice after 30 days of treatment in the group I-IV.
